# Rationale, Design and Participants Baseline Characteristics of a Crossover Randomized Controlled Trial of the Effect of Replacing SSBs with NSBs versus Water on Glucose Tolerance, Gut Microbiome and Cardiometabolic Risk in Overweight or Obese Adult SSB Consumer: Strategies to Oppose SUGARS with Non-Nutritive Sweeteners or Water (STOP Sugars NOW) Trial and Ectopic Fat Sub-Study

**DOI:** 10.3390/nu15051238

**Published:** 2023-02-28

**Authors:** Sabrina Ayoub-Charette, Néma D. McGlynn, Danielle Lee, Tauseef Ahmad Khan, Sonia Blanco Mejia, Laura Chiavaroli, Meaghan E. Kavanagh, Maxine Seider, Amel Taibi, Chuck T. Chen, Amna Ahmed, Rachel Asbury, Madeline Erlich, Yue-Tong Chen, Vasanti S. Malik, Richard P. Bazinet, D. Dan Ramdath, Caomhan Logue, Anthony J. Hanley, Cyril W. C. Kendall, Lawrence A. Leiter, Elena M. Comelli, John L. Sievenpiper

**Affiliations:** 1 Department of Nutritional Sciences, Temerty Faculty of Medicine, University of Toronto, Toronto, ON M5S 1A8, Canada; sabrina.ayoubcharette@mail.utoronto.ca (S.A.-C.); nema@emnhealth.com (N.D.M.); danie.lee@mail.utoronto.ca (D.L.); tauseef.khan@utoronto.ca (T.A.K.); sonia.blancomejia@mail.utoronto.ca (S.B.M.); laura.chiavaroli@alumni.utoronto.ca (L.C.); meaghan.kavanagh@utoronto.ca (M.E.K.); amel.taibi@utoronto.ca (A.T.); tzuhuan.chen@utoronto.ca (C.T.C.); amnaz.ahmed@mail.utoronto.ca (A.A.); madeline.erlich@mail.utoronto.ca (M.E.); yuetong.chen@mail.utoronto.ca (Y.-T.C.); vasanti.malik@utoronto.ca (V.S.M.); richard.bazinet@utoronto.ca (R.P.B.); anthony.hanley@utoronto.ca (A.J.H.); cyril.kendall@utoronto.ca (C.W.C.K.); lawrence.leiter@unityhealth.to (L.A.L.); elena.comelli@utoronto.ca (E.M.C.); 2 Toronto 3D Knowledge Synthesis and Clinical Trials Unit, Clinical Nutrition and Risk Factor Modification Centre, St. Michael’s Hospital, Toronto, ON M5C 2T2, Canada; 3 Sunnybrook Health Sciences Centre, Toronto, ON M4N 3M5, Canada; maxineseider@gmail.com; 4 Department of Chemical Engineering and Applied Chemistry, Faculty of Applied Science and Engineering, University of Toronto, Toronto, ON M5S 3E5, Canada; rachel.asbury@utoronto.ca; 5 College of Dietitians of Ontario, Ontario, ON M2M 4J1, Canada; 6 Department of Nutrition, Harvard T.H. Chan School of Public Health, Boston, MA 02115, USA; 7 Guelph Research and Development Centre, Science and Technology Branch, Agriculture and Agri-Food Canada, Government of Canada, Guelph, ON N1G 5C9, Canada; dan.ramdath@canada.ca; 8 College of Pharmacy and Nutrition, University of Saskatchewan, Saskatoon, SK S7N 5E5, Canada; 9 Nutrition Innovation Centre for Food and Health (NICHE), School of Biomedical Sciences, Ulster University, Co., Londonderry BT52 1SA, BT52 1SA Coleraine, Ireland; c.logue@ulster.ac.uk; 10 Division of Endocrinology and Metabolism, Dalla Lana School of Public Health, University of Toronto, Toronto, ON M5T 3M7, Canada; 11 Leadership Sinai Centre for Diabetes, Mount Sinai Hospital, Toronto, ON M5G 1X5, Canada; 12 Division of Endocrinology and Metabolism, Department of Medicine, St. Michael’s Hospital, Toronto, ON M5C 2T2, Canada; 13 Department of Medicine, Temerty Faculty of Medicine, University of Toronto, Toronto, ON M5S 1A8, Canada; 14 Li Ka Shing Knowledge Institute, St. Michael’s Hospital, Toronto, ON M5B 1T8, Canada

**Keywords:** low- and no-calorie sweeteners, sweetening agents, sugar-sweetened beverages, water, randomized controlled trial, type 2 diabetes, gut microbiota, glycemia, glucose control, overweight

## Abstract

Background: Health authorities are near universal in their recommendation to replace sugar-sweetened beverages (SSBs) with water. Non-nutritive sweetened beverages (NSBs) are not as widely recommended as a replacement strategy due to a lack of established benefits and concerns they may induce glucose intolerance through changes in the gut microbiome. The STOP Sugars NOW trial aims to assess the effect of the substitution of NSBs (the “intended substitution”) versus water (the “standard of care substitution”) for SSBs on glucose tolerance and microbiota diversity. Design and Methods: The STOP Sugars NOW trial (NCT03543644) is a pragmatic, “head-to-head”, open-label, crossover, randomized controlled trial conducted in an outpatient setting. Participants were overweight or obese adults with a high waist circumference who regularly consumed ≥1 SSBs daily. Each participant completed three 4-week treatment phases (usual SSBs, matched NSBs, or water) in random order, which were separated by ≥4-week washout. Blocked randomization was performed centrally by computer with allocation concealment. Outcome assessment was blinded; however, blinding of participants and trial personnel was not possible. The two primary outcomes are oral glucose tolerance (incremental area under the curve) and gut microbiota beta-diversity (weighted UniFrac distance). Secondary outcomes include related markers of adiposity and glucose and insulin regulation. Adherence was assessed by objective biomarkers of added sugars and non-nutritive sweeteners and self-report intake. A subset of participants was included in an Ectopic Fat sub-study in which the primary outcome is intrahepatocellular lipid (IHCL) by 1H-MRS. Analyses will be according to the intention to treat principle. Baseline results: Recruitment began on 1 June 2018, and the last participant completed the trial on 15 October 2020. We screened 1086 participants, of whom 80 were enrolled and randomized in the main trial and 32 of these were enrolled and randomized in the Ectopic Fat sub-study. The participants were predominantly middle-aged (mean age 41.8 ± SD 13.0 y) and had obesity (BMI of 33.7 ± 6.8 kg/m^2^) with a near equal ratio of female: male (51%:49%). The average baseline SSB intake was 1.9 servings/day. SSBs were replaced with matched NSB brands, sweetened with either a blend of aspartame and acesulfame-potassium (95%) or sucralose (5%). Conclusions: Baseline characteristics for both the main and Ectopic Fat sub-study meet our inclusion criteria and represent a group with overweight or obesity, with characteristics putting them at risk for type 2 diabetes. Findings will be published in peer-reviewed open-access medical journals and provide high-level evidence to inform clinical practice guidelines and public health policy for the use NSBs in sugars reduction strategies. Trial registration: ClinicalTrials.gov identifier, NCT03543644.

## 1. Introduction

Sugar-sweetened beverages (SSBs) are the single largest contributor of added sugars in the diet [1,2]. Health authorities are universal in discouraging the consumption of SSBs [2,3,4,5] as excess intake is associated with weight gain, type 2 diabetes, and non-alcoholic fatty liver disease (NAFLD) [6,7,8,9,10]. Non-nutritive sweetened beverages (NSBs), the greatest largest source of non-nutritive sweeteners (NNS) [11], provide a sweet alternative to SSBs without the calories. Health authorities are near universal in their recommendation that water preferentially replace SSBs. However, these health authorities are mixed in their recommendations regarding NSBs as a replacement strategy for SSBs with some recommending their use [12] and others, including Health Canada and the World Health Organization (WHO), recommending against their use over due to concerns that they have not demonstrated the intended benefits [5,13,14,15,16,17,18]. Whether NSBs are comparable to water as a replacement strategy for SSBs and reduce adiposity and adiposity-related non-communicable diseases (NCDs) is unclear. NNS mimic the taste of sugar, but are used in much smaller quantities [19], as they are much sweeter than sucrose [20]. The NNS that sweeten NSBs have been shown to be safe [20,21,22,23] and are approved for use in Canada [24] and regulatory authorities globally [25]. NSBs may be sweetened by a single NNS or NNS blends. For example, in Canada, Coke Zero, Diet Coke, Diet Pepsi, and Diet 7UP are sweetened by aspartame and acesulfame-potassium (ace-k), while Diet Orange Crush is sweetened by sucralose alone.

Recent comprehensive syntheses of the evidence of randomized controlled trials and prospective cohort studies have contributed to the uncertainty regarding NNS. A WHO-commissioned systematic review and meta-analysis of NNS concluded that most health outcomes showed no difference, meaning they failed to show the expected re-ductions in body mass index (BMI), body weight, plasma insulin levels, insulin resistance and β-cell function in randomized controlled trials (RCTs) or associations with lower risk of NCDs [26]. A second comprehensive systematic review and meta-analysis of RCTs showed that NNS did not result in the expected weight loss and were associated with higher risk of weight gain, type 2 diabetes, and cardiovascular disease in prospective cohort studies [27]. An important criticism of these systematic reviews and meta-analyses of RCTs has been their failure to account for the nature of the comparator and the calories displaced by NSBs, as caloric and noncaloric comparators were pooled together or non-caloric comparators were used as the sole comparator, which would lead to an underestimation of the effect of NSBs in their intended substitution for SSBs [28,29,30,31,32]. When analyses were restricted to comparisons with SSBs (allowing for caloric displacement), there were the expected decreases in blood glucose levels and blood pressure in these same syntheses [26,27]. These evidence syntheses also did not differentiate between the food sources that contain NNS, in which it can be difficult to achieve differences in energy as gram amounts of sugars are replaced with milligram amount of NNS with starches and fats as fillers making up the difference by weight [26]. Epidemiologic analyses have proven equally problematic, as baseline or prevalent exposure of NNS and NCD outcomes is well recognized to be at high risk of reverse causality and residual confounding from behavioral clustering [29,31,32,33]. More recent systematic reviews and meta-analyses designed to address these criticisms by looking specifically at the substitution of NSBs for SSBs as a comparison of a single food matrix and the most important source of NNS (i.e., NSBs) and free sugars (i.e., SSBs) in the diet. These systematic reviews and meta-analyses showed the expected differences in adiposity and adiposity-related markers in both the randomized controlled trials [16,34,35,36,37] and prospective cohort studies [38]. These syn-theses reinforced the importance of energy displacement and food matrix and support the shift in dietary guidelines from a focus on single nutrients towards a focus on foods, in recognition that focus on single nutrients may miss important interactions related to the food matrix in which the nutrients are contained [39].

Various biological mechanisms have been invoked to support the signals for concern regarding NNS consumption (e.g., through the brain’s reward center [40], and/or through gastrointestinal sweet-taste receptors [41]), but there is a particular concern that NNSs may induce and promote glucose intolerance through changes to the composition and diversity of the gut microbiome [30,42]. A single study showed a worsening of glucose tolerance and changes in gut microbiota beta-diversity in a post-hoc “responder” group after six days of saccharin capsules administration at the maximum acceptable daily intake (ADI) dose (5 mg/kg of body weight/day) [43]. Despite this study’s methodological weaknesses (short duration, small sample size, before-and-after design etc.), it reinforced a negative view of NNSs in the media [44,45,46,47,48,49,50,51]. More recent intervention trials designed to address the gaps described have failed to confirm these initial results [42,52,53,54], using various single NNS (aspartame, sucralose, and saccharin) in healthy normal weight adults on markers of glycemic control and gut microbiota beta-diversity. However, there is still controversy in this field, as a 2022 study showed a worsening of glucose tolerance and changes in gut microbiota beta-diversity in a different post-hoc “responder” group after 14 days of saccharin and sucralose sachet administration at 34 and 20% of the ADI dose, respectively [55]. These studies still leave many pragmatic questions unanswered such as, dose- and time-dependent effects, the effect of the most commonly consumed NNS and NNS blends (which is the most common way that blends being the most common way NNS are consumed worldwide [25], especially since different NNS may have different physio-logical effects) and in the food sources which they are mostly found (i.e., NSBs) [56,57]. More importantly, none of these studies answered the question of the intended use of NNS, which is to displace calories from sugars, particularly from SSBs for disease prevention. 

There is an urgent need to address the remaining uncertainties related to NSBs. Health Canada, in particular, has indicated that studies of sugar reduction strategies that use NNSs with microbiome outcomes are an important research priority [58]. We undertook the Strategies To OPpose SUGARS with Non-nutritive sweeteners Or Water (STOP Sugars NOW) trial, a Canadian Institutes for Health Research (CIHR)-funded randomized controlled trial to assess the effect of a “real world” strategy of SSBs reduction using NSBs on glucose tolerance and gut microbiota changes as well as intermediate cardiometabolic risk factors and mediators. We also undertook a sub-study on liver fat and related ectopic muscle fat and intermediate NAFLD outcomes (Ectopic Fat sub-study), given the recognition of NAFLD as an early metabolic lesion in the pathogenesis of type 2 diabetes and an increasingly common public health problem [59]. To address the issue of the nature of the comparator, we designed a study to assess NSBs in the context of three prespecified substitutions of clinical and public health concern: NSBs for SSBs (“intended substitution” with caloric displacement), water for SSBs (“standard of care substitution” with caloric displacement) and NSBs for water (“reference substitution” without caloric displacement).

## 2. Materials and Methods

### 2.1. Trial Design

The STOP Sugars NOW trial is a four-week single-center, open label, randomized controlled crossover trial with three arms (SSB, NSB, water) comparing the effect of replacing SSBs with NSBs (“intended replacement”) versus water (“standard of care”) on the gut microbiota diversity and glucose tolerance. After a two-week run-in phase, each participant acted as their own control receiving the three interventions for four-weeks each in a random order, with intervention phases separated by a minimum of a four-week washout phase. All trial visits were conducted at Unity Health Toronto, St. Michael’s Hospital, Toronto, ON. Canada. The trial protocol conforms to the ethical guidelines of the Tri-Council Policy Statement 2 [60], and the study was conducted according to the guidelines of the Declaration of Helsinki and approved by the research ethics board of St. Michael’s Hospital (protocol code: 17-292, 16 February 2018). All participants provided written informed consent to the main trial, and separately but optional, to the Ectopic Fat sub-study. The trial is registered on ClinicalTrials.gov (NCT03543644), and Supplemental File S1 includes the full trial protocol. All methods described here apply for the Ectopic Fat sub-study except where indicated.

### 2.2. Inclusion and Exclusion Criteria

Table 1 shows the detailed list of inclusion and exclusion criteria. Participants were included if they were consuming at least one 355 mL serving of SSB per day. Additional inclusion criteria include: between the ages of 18 and 75 years, BMI that is classified as overweight or obese (BMI ≥ 23 kg/m^2^ for Asian individuals and ≥25 kg/m^2^ for other individuals), and high waist circumference (using ethnic specific cut-offs [61,62,63,64]) but otherwise healthy with no antibiotic use in the last three months [65,66,67,68,69]. Main exclusion criteria were if they were pregnant or breastfeeding or planning on becoming pregnant during the trial and if they had any disease, among others. The Ectopic Fat sub-study followed the same inclusion and exclusion criteria with the addition of one factor for exclusion: any condition or circumstance which would prevent from having an ^1^H-MRS scan (e.g., having prostheses or metal implants, tattoos, or claustrophobia).

### 2.3. Randomization and Allocation Concealment

Table 2 shows the Latin square randomization table. Randomization was performed after successful completion of the run-in phase and first visit. Randomization, with no stratification, was conducted centrally by an offsite statistician at the Applied Health Research Centre (AHRC) at St. Michael’s Hospital using the Research Data Capture (REDCap) program. Participants were randomly allocated to six possible sequences using blocked (Latin squares) randomization with a similar number of participants allocated to each treatment sequence. Allocation concealment was achieved by the secured electronic delivery of a single sequence for each consecutive participant. RedCap was chosen as it exceeds privacy and security standards which enables anonymization, secure information storage, retrieval, and sharing of data.

### 2.4. Interventions

Table 3 shows the list of intervention beverages and types of NNS sweeteners available for the trial. There were three interventions: SSBs (355 mL, 140 kcal, 42 g sugars per serving); NSBs (355 mL, 0 kcal, 0 g sugars per serving); and water (still or carbonated) (355 mL, 0 kcal, 0 g sugars per serving). To allow for pragmatic substitutions using available market products, the calories of the intervention groups were not matched, and the dose prescription of each intervention (number of 355 mL servings) for each participant was matched to their baseline SSB intake. Participants were provided with the SSB of their choice, equivalent NSB that was sweetened by either acesulfame-potassium (ace-k) or sucralose, as these will be measured objectively as markers of adherence (see below) [71], or water. If participants were consuming NSBs in addition to SSBs, they forwent their NSBs. The beverages given to participants during the trial were not necessarily selected to be in the same lot.

### 2.5. Blinding

Blinding of the participants and trial personnel was not possible due to the nature of the intervention. However, outcome assessors and statisticians were blinded to the identity of the treatments.

### 2.6. Trial Flow

**Figure 1:**
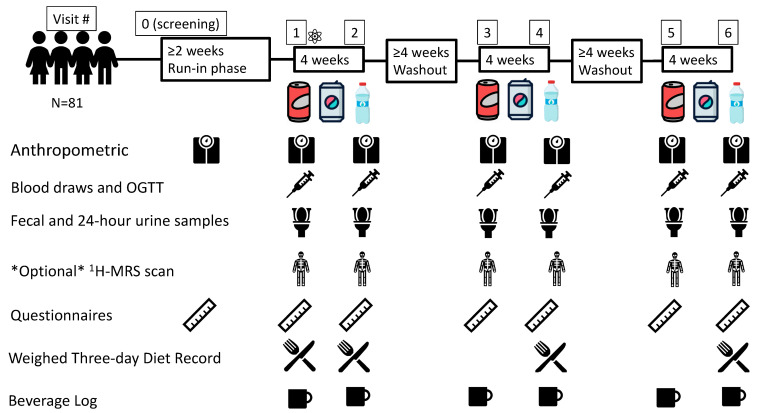
Flow of Participants through the Trial and the Data Collection Schedule. Questionnaires include Personal Information, Demographic Data and Medical History Questionnaires, Symptoms and Craving/Hunger/Satiety Questionnaires and Case Report Forms. 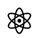 = Randomization event: AHRC-REDCap program; Blocked (Latin squares) randomization; Allocation concealment; 1H-MRS = proton magnetic resonance spectroscopy; 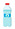 = Water; 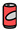 = sugar-sweetened beverage (SSB); 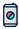 = non-nutritive sweetened beverage (NSB); N = number; OGTT = 2-h 75 g oral glucose tolerance test.

Figure 1 shows the flow of participants through the trial and the data collection schedule. Participants were recruited through postings and flyer handouts, transit advertisements, online listings (Craigslist and Kijiji) and through a digital marketing group (Trialfacts). After informed consent review and in-person screening, eligible participants who consented to participate in the trial were instructed on data collection procedures and were enrolled in a minimum two-week run-in phase.

After randomization and for the trial duration, participants were asked to maintain their usual background diet and exercise regimen. Before every visit, participants were instructed to collect a whole stool sample, a 24 h urine sample, and a weighed three-day diet record (3DDR); to consume a minimum of 150 g of carbohydrate for each of the three days prior; and to arrive fasted for 10–12 h [72,73]. Common examples of 150 g of carbohydrates were shared with participants prior to their visit (e.g., 2–3 slices of bread, 1 cup of cooked rice/pasta, 1 medium potato, etc.). At each trial visit, if participants were enrolled in the Ectopic Fat sub-study, they complete an ^1^H-MRS scan. Then, the standard protocol was followed for the administration of a 2 h 75 g oral glucose tolerance test (OGTT) (time points −30, 0, 30, 60, 90, 120) [74], which was followed by breakfast prepared by the study staff.

Once all measures were collected, participants were provided the intervention beverages and beverage log sheets, urine and stool containers and instructions for their next visit. After successful randomization, participants were compensated for their travel expenses and for their time. Participants who were lost to follow-up were compensated for each trial visit that was completed.

The four-week duration of each intervention phase was chosen to allow changes in our family of primary outcomes (glucose tolerance and gut microbiota diversity), as previous studies have seen changes in microbiota diversity with seven days to two weeks intervention [43,55]. To control for any carry-over effects of one beverage type over another, each of the three intervention phases was separated by a minimum four-week washout phase where participants reverted to their regular SSB intake. Participants were given beverage logs to complete over the washout. Antibiotic use during a washout or intervention phase required either prolongation of the washout period or stoppage of the intervention phase followed by a minimum 30-day washout period measured from the time of completion of the antibiotic course [69].

To promote adherence, all intervention beverages were provided either by pick-up by the participant or by home delivery; participants completed beverage log sheets and motivational phone calls and emails were made every two weeks.

### 2.7. Primary Outcomes

The family of primary outcomes of the main trial includes changes from baseline in oral glucose tolerance, as measured by the glucose incremental area under the curve (iAUC), and the gut microbial beta-diversity, as measured by the weighted UniFrac distance matrix. The weighted UniFrac distance matrix was chosen as it was found to be more accurate [75]. This will be computed using the QIIME2 [76] pipeline with DNA sequences (sequenced using the Illumina MiSeq platform) from the 16S *rRNA* gene, with primers targeting the V3–V4 region. The QIIME2 pipeline will be available on GitHub upon request.

The primary outcome of the Ectopic Fat sub-study is changes from baseline in intrahepatocellular lipid (IHCL), as measured by ^1^H-MRS.

### 2.8. Secondary Outcomes

The secondary outcomes of the main trial are changes from baseline in waist circumference, body weight, fasting plasma glucose (FPG), 2 h plasma glucose (2 h-PG) and the Matsuda whole body insulin sensitivity index (Matsuda ISI_OGTT_) [77].

The secondary outcomes of the Ectopic Fat sub-study are change from baseline in intramyocellular lipid (IMCL), fatty liver index (FLI) [78], alanine aminotransferase (ALT), aspartate aminotransferase (AST), glutamyl transferase (GGT) and alkaline phosphatase (ALP).

### 2.9. Adherence Outcomes

Adherence outcomes are changes from baseline in self-reported beverage intake from beverage logs, returned beverage containers, and objective biomarkers of SSBs (increased 13C/12C ratios in serum fatty acids [79] and increased urinary fructose [80]), water (decreased 13C/12C ratios in serum fatty acids [79] and decreased urinary fructose [80]), and NSB (increased urinary acesulfame potassium and/or sucralose [71]) intake.

### 2.10. Exploratory Outcomes

Exploratory outcomes of the main trial include changes from baseline in blood pressure, fasting blood lipid profile, fasting plasma insulin, 75 g OGTT derived indices (iAUC plasma insulin, maximum concentrations (C_max_) and time to maximum concentrations (T_max_)) of plasma glucose and insulin, mean incremental plasma glucose and insulin, the Homeostatic Model Assessment for Insulin Resistance (HOMA-IR) [81,82], beta-cell function as measured by the insulin secretion-sensitivity index-2 (ISSI-2) [83,84], early insulin secretion index [85,86], satiety, hunger, and food cravings, diet quality, alpha-diversity, other beta-diversity indexes and metagenomic inference from compositional data in silico from whole stool [87].

### 2.11. Power Calculation

Table 4 shows the power (sample size) calculation for the main trial and the Ectopic Fat sub-study, using the “power” package in STATA17 (StataCorp, College Station, TX, USA). The main trial has over 89% power and the Ectopic Fat sub-study has over 80% power to show a difference between the NSB, water and SSB arms in 60 participants in the two primary outcomes of glucose tolerance and gut microbiota beta-diversity in the main trial and 25 participants in the primary outcome of IHCL in the Ectopic Fat sub-study. Assuming a drop-out rate of 20%, we planned to recruit 75 and 32 participants, respectively.

The main trial is powered to detect a difference of 44.81 mmol/L/min (standard de-viation (SD) = 113 mmol/L/min) (i.e., 20%) in iAUC glucose [88,89] and 0.04 (SD = 0.07) in microbiota beta-diversity [43]. Considering it is a cross-over trial with a within-person correlation of 0.7, these differences were chosen based on effects smaller than the be-ta-diversity and glycemic response effect observed in the study of Suez et al. (2014) [43], which was the only study available at the time. The trial is also powered to detect clinically meaningful differences in all secondary outcomes except the adiposity outcomes (body weight and waist circumference) between the three interventions [35,90,91]. To control for false discovery, the truncated Benjamini-Hochberg method with parallel gatekeeping for control of false discovery will be implemented [93,94,95,96]. By this method the unused portion of the alpha from the primary outcomes is passed onto the secondary family of outcomes if either one of the primary outcomes is statistically significant. The sample size calculations were based on the most conservative alpha estimates from this method. If none of the primary endpoints reaches significance, then the secondary outcomes will be analyzed as exploratory variables without adjustment for false discovery rate. All exploratory out-comes will be assessed without adjustment for false discovery rate. 

The Ectopic Fat sub-study is powered to detect a difference of 5% (SD = 10%) in IHCL [92], using a within-person correlation of 0.67. The truncated Benjamini-Hochberg method with parallel gatekeeping procedure will not be used for the Ectopic Fat sub-study, as it is considered exploratory in nature.

### 2.12. Outcome Assessment

Supplementary File S1 presents the methods for the assessment of the primary, secondary, adherence, and exploratory outcomes.

### 2.13. Statistical Analysis

Data will be analyzed in STATA 17 (StataCorp, College Station, TX, USA). Primary analyses will be according to intention-to-treat (ITT) principle with multiple imputations or other appropriate statistics. Additional prespecified analyses will be undertaken that include completers and the per-protocol analyses. Repeated measures mixed effect models will be used to assess changes in the family of primary outcomes (gut microbiota beta-diversity (weighed UniFrac distance) and mean glucose iAUC (75 g OGTT)) between the groups and in our secondary outcomes (waist circumference, body weight, FPG, 2 h-PG and Matsuda ISI_OGTT_) with adjustment for sequence effects, trial completion during the coronavirus disease 2019 (COVID-19) pandemic, withdrawal/drop-out during the COVID-19 pandemic, and antibiotic use during the trial. Other adjustments will be considered based upon an assessment of any imbalances during the trial. Pairwise comparisons will be performed using Tukey–Kramer adjustment to assess differences for the three prespecified substitutions: SSBs for NSBs (“intended substitution” with caloric displacement), SSBs for water (“standard of care substitution” with caloric displacement) and NSBs for water (“reference substitution” without caloric displacement). We will use the truncated Benjamini–Hochberg false discovery rate controlling method with a parallel gatekeeping procedure to correct for multiple outcomes for all the primary and secondary endpoint comparisons in the main analysis as described above [93,96]. To assess effect modification, a priori subgroup analysis will be conducted by age, sex, ethnicity, antibiotic use during the trial, baseline BMI, baseline waist circumference, baseline FPG, baseline 2 h-PG, baseline iAUC, medication use, NNS blend consumed from trial beverages in the NSB arm, SSB type and background NNS use. Subgroup analyses by baseline SSB dose (as number of 355 mL serving per day and percent energy from sugars), consumption patterns, energy compensation, trial completion during the COVID-19 pandemic and caffeine intake (cola vs. non-cola beverages) that have emerged as relevant prior to data analyses will also be considered.

## 3. Results

### 3.1. CONSORT Statement

**Figure 2:**
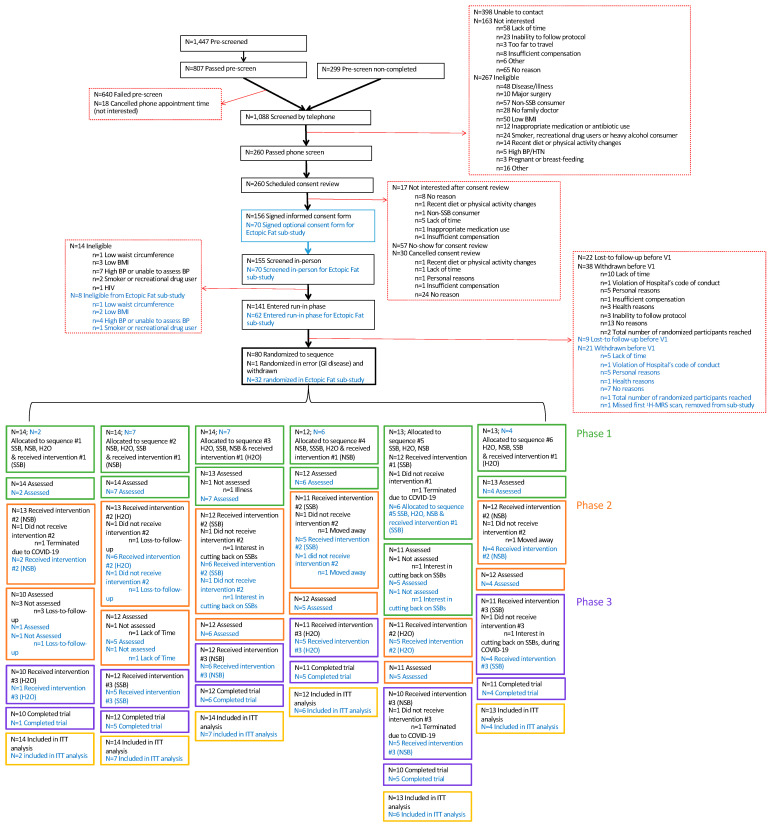
Flow of Participants through the Trial and the Data Collection Schedule. Text in blue refers to the Ectopic Fat sub-study. ^1^H-MRS= proton magnetic resonance spectroscopy; BMI = body mass index; BP = blood pressure; COVID-19 = coronavirus disease 2019; GI = gastrointestinal; H2O = water; HIV = human immunodeficiency virus; HTN = hypertension; ITT = intention-to-treat; N = number; NSB = non-nutritive sweetened beverage; SSB = sugar-sweetened beverage; V = visit; WC = waist circumference.

Figure 2 shows the CONSORT statement for the main trial and the Ectopic Fat sub-study. Enrollment began on 1 June 2018, with the first participant undergoing randomization on 22 November 2018. The last participant finished the Ectopic Fat sub-study on 18 February 2020 (prior to the start of the COVID-19 pandemic). The main trial was interrupted owing to research closures at St. Michael’s Hospital due to the COVID-19 pandemic between March 2020 and September 2020, which resulted in visits being halted for 10 participants. During this time, participants were placed on a washout period pending the restart of research; any intervention that needed to be stopped prior to completion was restarted from baseline. The trial resumed with the permission of the Research Ethics Board in September 2020 with completion of the last trial participant on 15 October 2020.

The planned recruitment was expanded from 75 to 81 participants for the main trial and from 25 to 32 for the Ectopic Fat sub-study to increase the power for the planned analyses. The increase in power was approved before the COVID-19 pandemic and was completed to allow participants already enrolled in the run-in phase to have a chance to participate.

A total of 1088 individuals completed the telephone screening questionnaire, of which 260 were eligible for in-person screening and 156 provided written informed consent. Main reasons for the failed screening included failure to contact (n = 398), lack of interest (n = 163) and ineligibility (n = 267) mainly due to non-consumption of SSBs (n = 57) or the presence of disease (n = 48). After the in-person screening, 141 individuals were eligible for the main trial, of which 62 individuals consented to the Ectopic Fat sub-study. Due to dropouts (main trial = 38; Ectopic Fat sub-study = 21) and loss-to-follow up (main trial = 22; Ectopic Fat sub-study = 9) during the screening and run-in phases, a total of 81 eligible participants (Ectopic Fat sub-study = 32) were randomized to a treatment sequence. One participant met the exclusion criterion for the presence of gastrointestinal disease, was randomized in error to the main trial and was withdrawn shortly after randomization. Their randomization sequence was not reused, and the participant was not counted among the randomized participants and will not be included in any analyses. A total of 66 participants out of 80 completed the main trial (83% retention), and 26 out of 32 participants completed the Ectopic Fat sub-study (81% retention). Of the 10 participants who were enrolled in the main trial during the COVID-19 pandemic, one participant dropped out during the pandemic (due to changes in lifestyle (interest in cutting back on SSB consumption)), three participants who had more than one phase to complete were withdrawn by the investigators (as it was determined there was no reasonable prospect of their completion during the projected subsequent waves of COVID-19), and the remaining six participants who had only a single phase to complete were able to return and finish the trial. The reasons for attrition unrelated to the COVID-19 pandemic ranged from lack of time to participate, moving away, change in lifestyle (cutting back on SSB consumption) or loss-to-follow-up.

### 3.2. Baseline Characteristics

Table 5 shows the baseline characteristics of the 80 randomized participants in the main trial and the 32 participants randomized in the Ectopic Fat sub-study participants. Baseline characteristics are descriptively described here: Mean (SD) age was 42.34 years (12.99 years) (Ectopic Fat sub-study: 42.16 years (12.91 years)), with approximately even sex distribution (main trial, female = 51%; Ectopic Fat sub-study, female = 50%). BMI and waist circumference were 33.71 kg/m^2^ (6.75 kg/m^2^) (Ectopic Fat sub-study, 33.70 kg/m^2^ (6.03 kg/m^2^)) and 108.69 cm (13.50 cm) (Ectopic Fat sub-study, 110.31 cm (13.67 cm)), respectively. The mean baseline (SD) systolic and diastolic blood pressure were 116.37 mmHg (12.49 mmHg) (Ectopic Fat sub-study: 76.68 mmHg (9.12 mmHg)) and 76.24 mmHg (9.03 mmHg) (Ectopic Fat sub-study, 72.34 mmHg (9.76 mmHg)), respectively. Mean baseline (SD) FPG was 5.57 mmol/L (1.19 mmol/L) (Ectopic Fat sub-study: 5.77 mmol/L (1.75 mmol/L)) and 2 h-PG were 7.26 mmol/L (3.11 mmol/L) (Ectopic Fat sub-study: 7.99 mmol/L (4.05 mmol/L)). Mean baseline (SD) IHCL for the participants in the Ectopic Fat sub-study was 9.7% (9.2%). Most trial participants are of European (main trial: 45%; Ectopic Fat sub-study, 56%) descent. About one-third of participants in the main trial had achieved an undergraduate education (34%), whereas a similar proportion of participants in the Ectopic Fat sub-study had a high school diploma (31%). About half of the participants worked full-time (main trial: 50%; Ectopic Fat sub-study: 44%), and one-quarter of participants in the main trial reported drinking no alcohol (26%), with only 4% drinking alcohol daily. Among the Ectopic Fat sub-study, a similar proportion reported drinking alcohol once every 2–3 months (28%) with only 6% drinking alcohol daily.

Overall, 29% and 38% of participants in the main trial and Ectopic Fat sub-study, respectively, reported being on regular medications. Some participants reported taking supplements (main trial: 29%; Ectopic Fat sub-study: 25%). Two participants (9%) included in the main trial (one (13%) in the Ectopic Fat sub-study) used marijuana regularly without a medical prescription.

Most participants did not regularly consume NNS in the past six months before baseline (main trial: 55%; Ectopic Fat sub-study: 63%), while some participants reported background NNS intake from beverages (main trial: 26%; Ectopic Fat sub-study: 31%), table-top added sweeteners (main trial: 6%; Ectopic Fat sub-study: 3%), foods (main trial: 5%; Ectopic Fat sub-study: 0%), or a mix of these sources (main trial: 8%; Ectopic Fat sub-study: 3%). Baseline SSB preference revealed Coca-Cola (main trial: 44%; Ectopic Fat sub-study: 53%) as the most popular SSB consumed, with an overall average of 1.84 servings (355 mL) per day (total intake: 653.2 mL/d) in the main trial and 2.02 servings (355 mL) (total intake: 717.1 mL/d) per day in the Ectopic Fat sub-study. From this information, projected NSB equivalent intake indicated that an overwhelming majority of participants consumed a blend of aspartame and ace-k (main trial: 95%; Ectopic Fat sub-study: 94%) during the NSB phase. Overall, baseline characteristics in the Ectopic Fat sub-study were descriptively similar to those in the main trial.

## 4. Discussion

The STOP Sugars NOW Trial is a pragmatic, single-center, open label, randomized controlled multiple crossover trial with three four-week treatment phases (SSB, NSB, water) comparing the effect of the substitution of NSBs (“intended substitution”) versus water (“standard of care substitution”) for SSBs on the gut microbiota and glucose tolerance as well as other intermediate cardiometabolic outcomes in adults who consume ≥1 SSBs daily and are overweight or obese with a high waist circumference. The Ectopic Fat sub-study uses the same design to assess the effect on ectopic fat in liver (IHCL) by ^1^H-MRS as well as other ectopic fat in muscle (IMCL) and related intermediate markers of NAFLD. This trial will be the first to investigate the effect of the intended use of NSBs to replace SSBs, compared with the “standard of care” water, in a pragmatic and controlled manner in those who are regular consumers of SSBs and at high risk of the sequelae of overconsumption of SSBs.

Baseline characteristics for both the main and Ectopic Fat sub-study were not different. Overall, the study participants in both the main trial and the Ectopic Fat sub-study represent an overweight or obese group with characteristics putting them at risk for type 2 diabetes and other NCDs.

The NNS tested in the present trial are representative of those available on the market in Canada and globally [97]. Consumption of NSBs has been increasing [11,98] with approximately 10% of Canadians consuming NSBs [99]. Canadian market share data show that Diet Pepsi, Diet Coke and Coke Zero are the leading NSB brands in Canada [100]. As NSBs are the most important food source of NNS, it can be inferred that the blend of aspartame and ace-k, followed by sucralose alone, are the most common NNS in foods in Canada, and this data reflects the global market [97]. 

### 4.1. Strengths and Limitations

The present trial overcomes several limitations of previous trials of the use of NSBs as a replacement strategy for SSBs. First, it uses a “real-world” fully pragmatic design with NSBs products available on the Canadian and global markets [56,100], compared with previous work that mostly administered single NNS in capsule form and often in greater amounts than products available on the market, which limits the generalizability of conclusions [42,43,52,53,54]. Second, comparing the effect of NSBs to SSBs (“intended sub-stitution”) and the standard of care water (“reference substitution”) allows for the dis-entanglement of the effect of energy from that of the NNS, as it has been hypothesized that NNS may have consequences for health independent of energy content that result from the chemical compounds themselves [41,56,101]. The substitution of NSBs for SSBs allows for the displacement of energy, which is often overlooked in controlled trial syntheses, including a recent WHO-commissioned review [26]. The substitution of water for SSBs (“standard of care substitution”) and comparison of water with NSBs (“reference substitution”), will clarify whether NSBs are like water in their effect on gut microbiota and attributing metabolic disease risk. Third, unlike many studies that have investigated changes in the gut microbiota beta-diversity as an outcome [102], the STOP Sugars NOW trial is powered to detect a change in both primary outcomes of glucose tolerance and change in the beta-diversity of the gut microbiota, using Food and Drug Administration recommended statistical analyses [96]. Fourth, our use of objective urinary and serum biomarker analysis to assess adherence to all interventions overcomes many reporting or recall biases. Fifth, our sample population that includes participants who are overweight or obese with a high waist circumference represents an at-risk population for type 2 diabetes. This makes the results of the trial relevant to guidelines and policies for type 2 diabetes prevention. Sixth, the results from this trial will contribute to the development of clinical trial methodologies, especially related to the integration of microbiota data with clinical data, which is an emerging area of research [103]. Finally, our results will be directly translatable to most areas of the world which share similar NNS and NSB availability. 

Some limitations remain. First, the multiple crossover design with three treatment phases, two washout periods, and multiple measurements may have contributed to a reduction in retention. Our retention of 82.5% for the main trial and 81.3% for the Ectopic Fat sub-study, however, would meet criteria for good retention [104]. We also anticipated this level of retention in our sample size calculation and have been able to maintain adequate statistical power for our primary and secondary outcomes with good balance in the treatment sequences. Second, as with any intervention trial, participants may un-consciously compensate for the reduced energy and caffeine intake from NSBs or water interventions by consuming additional calories in their background diet [34]. Despite instructions to maintain usual background diets and activity levels throughout the trial, the inventions may also have engendered other health behaviors leading participants to change their diets and activity levels consciously or unconsciously [105]. As part of the ITT principle, any changes resulting from the interventions would be considered an effect of the interventions and will be captured in our analysis of the participant’s 3DDRs, activity logs, and case report forms, and assessed by subgroup analyses. The design which included both negative (SSBs) and positive (water) controls will also help us to isolate the effect of the change in NSBs from other changes which may attenuate or enhance the effects of the interventions. Further, we will be conducting subgroup analysis to explore the effects of caffeine on our outcomes. Third, as most of the participants in the NSB phase consumed a blend of aspartame and ace-k, we will not be able to isolate the effect of a single NNS on glucose tolerance and the gut microbiota. Related to this limitation, our results will not inform the effect of other less-common NNS, such as neotame or saccharin, as these NNS are not used to sweeten commercially available NSBs in Canada. These results, however, will be representative of the product availability in the Canadian, North American, and global market [56,97,100]. Fourth, although our trial sample shows traits akin to the general population of Canada where the prevalence of overweight and obesity is now 63% [106,107] and akin to average SSB consumers in North America [10,11,108], we do acknowledge that there is some evidence of volunteer bias in our sample, as most of our participants represent higher socioeconomic background (higher education and full-time work status). Therefore, we are potentially neglecting inclusion of people who are at higher risk for type 2 diabetes and other NCDs, and who have less access to resources to manage the disease [109,110]. Fifth, the COVID-19 pan-demic posed some challenges. Ten participants were enrolled in the trial during the COVID-19 pandemic, of which six completed the last phase of the trial after a prolonged washout period, three were withdrawn by the investigators for no reasonable prospect of completion, and one dropped out during the pandemic due to changes in lifestyle. It is reasonable to expect that the background diet and lifestyle of these participants and their ability to adhere to the interventions and trial protocol were directly affected by the pandemic. It was decided to adjust for study participation during the pandemic in our primary models and conduct sensitivity analyses in which these participants were excluded in modified ITT, completers, and per protocol analyses. Sixth, the washout period may have been of insufficient duration and may result in carry-over effects from previous the intervention, especially for changes in the gut microbiota. However, the microbiome is very resilient; acute changes in diet or lifestyle revert to baseline within 48 hours [111]. Short-term dietary changes, especially of only one component of the diet, is often not sufficient to majorly perturb the gut microbiome in a permanent way [112]. The STOP Sugars NOW Trial is changing only one aspect in our participant’s diets for a short-term intervention: sugar-sweetened beverage intake for four weeks. During the washout phases, participants were instructed to revert to their usual sugar-sweetened beverage intake, and we believe that the resiliency of the gut microbiome will overcome the short-term dietary changes. Finally, as we are using 16S rRNA gene sequencing to assess gut microbiota outcomes, we will not have species level resolution, and we will not be able to directly infer gut microbiota functions. However, previous research done on the effect of non-nutritive sweeteners on the gut microbiota and the risk for diabetes [42,43,52,53,54], have also sequenced the 16S rRNA gene. Therefore, our results will be directly comparable to the current literature on this topic.

### 4.2. Implications

The role of NSBs in modifying the risk for type 2 diabetes from SSBs and the extent to which the human gut microbiota might mediate this process is of great importance in understanding chronic disease pathogenesis, prevention, and management for guideline and policy makers. Excess intake of calories from sugars, particularly from SSBs, has been linked to the rise in NCDs [6,7,8,9]. Public health agencies in Canada and around the world have responded by urging the public to reduce their consumption of SSBs [2,113,114,115]. Replacement strategies for SSBs that leverage beverages with the same sweet taste, like NSBs, may be more appealing than water and thus may facilitate a reduction in SSB intake. Although the NNSs used to sweeten NSBs are considered safe by regulatory authorities [22,23], public health recommendations are conflicting [2,18,114,116]. Some research suggests that NNS may increase the risk for type 2 diabetes through changes in the microbiota in humans, but this finding needs confirmation [42,88]. The proposed STOP Sugars NOW trial will provide important data to address this concern using representative NSBs and NNS blends in a representative sample of at-risk obese individuals. 

## 5. Conclusions

The STOP Sugars NOW trial is the first to pragmatically investigate the effect of the intended use of NSBs to replace SSBs compared with the “standard of care” water in those who are regular consumers of SSBs and at high risk of the sequelae of overconsumption of SSBs. We successfully recruited and randomized 80 participants in the main trial and 32 participants in the Ectopic Fat sub-study. Baseline characteristics for both the main and Ectopic Fat sub-study were similar and meet our eligibility criteria. Overall, the study participants in both the main trial and the Ectopic Fat sub-study represent an overweight or obese group with characteristics putting them at risk for type 2 diabetes and other NCDs. Additionally, the study sample is representative of the average SSB consumer in Canada, making the study’s results applicable to disease prevention in target population for public health interventions to reduce SSB intake. The results of this unique trial will be presented at scientific meetings, published in peer-reviewed academic journals, and included in updated systematic reviews and meta-analyses. The results will inform guidelines for NSBs as a replacement strategy for SSBs, compared with “standard of care” (water), aiding in knowledge translation related to the health effects of NSBs in their intended substitution for SSBs; improving health outcomes by educating healthcare providers and patients, stimulating industry innovation; and guiding future research design.

## Figures and Tables

**Table 1:** Inclusion and Exclusion Criteria.

Inclusion Criteria	Exclusion Criteria
Age between 18 and 75 years	Age below 18 years and greater than 75 years
Regularly consumes SSBs (≥1355 mL serving per day) ^2^	BMI < 23 kg/m^2^ for Asian individuals and <25 kg/m^2^ for other individuals ^1^
BMI ≥ 23 kg/m^2^ for Asian individuals and ≥25 kg/m^2^ for other individuals ^1^	Waist circumference <94 cm in men, <80 cm in women in Europid, Sub-Saharan African, Eastern Mediterranean, and Middle Eastern individuals; <90 cm in men and <80 cm in women for South Asian, Chinese, Japanese, and South and Central American individuals ^1^
Waist circumference >94 cm in men, >80 cm in women in Europid, Sub-Saharan African, Eastern Mediterranean, and Middle Eastern individuals; >90 cm in men and >80 cm in women for South Asian, Chinese, Japanese, and South and Central American individuals [61,62,63,64] ^1^	Not regularly drinking SSBs (<1355 mL can serving per day) ^2^
Otherwise healthy	Pregnancy or breast-feeding females, or females planning on becoming pregnant throughout the study duration
Has a primary care physician	Regular medication use that have a clinically relevant effect on the primary outcomes (exceptions include birth control and PRN meds such as Advil, Tylenol, etc.)
	Antibiotic use in the last 3 months [65,66,67,68,69]
	Use of CAM deemed inappropriate by investigators
	Diabetes
	Self-reported hypertension or systolic blood pressure ≥ 160 mmHg or diastolic blood pressure ≥ 100 mmHg [70] when measured at screening visit ^1^
	Polycystic ovarian syndrome
	Cardiovascular disease
	Gastrointestinal disease
	Previous bariatric surgery
	Liver disease
	Hyper-or hypothyroidism
	Kidney disease
	Chronic infection
	Lung disease
	Cancer/malignancy
	Schizophrenia spectrum and other psychotic disorders, bipolar and related disorders, and dissociative disorders
	Major surgery in the last 6 months
	Other major illness or health-related incidence within the last 6 months
	Regular cigarettes smoker
	Regular recreational drug users
	Heavy alcohol users (>3 drinks/day)
	Does not have a primary care physician
	Participation in any trials within the last 6 months or planning on participating in other trials for the duration of this study
	Plans to make dietary or physical activity changes throughout study duration
	Any condition or circumstance which would prevent you from having an ^1^H-MRS scan (e.g., having prostheses or metal implants, tattoos, or claustrophobia) (this is only applicable for the 32 participants in the Ectopic Fat sub-study)

All criteria are self-reported unless otherwise indicated. ^1^ Not self-reported. ^2^ SSBs include sodas and soft drinks that contain at least 50 kcal per 8-oz serving. They do not include fruit drinks or 100% fruit juice, sports, or energy drinks, sweetened iced tea or coffee, or homemade SSBs such as frescas or juices. 1H-MRS scan = proton magnetic resonance spectroscopy; BMI = body mass index; CAM = complementary or alternative medicine; SSBs = sugar-sweetened beverages; PRN = pro re nata.

**Table 2:** Latin Square Randomization Table.

	Intervention Phase 1	Intervention Phase 2	Intervention Phase 3
Sequence Group 1	A	B	C
Sequence Group 2	B	C	A
Sequence Group 3	C	A	B
Sequence Group 4	B	A	C
Sequence Group 5	A	C	B
Sequence Group 6	C	B	A

Rows represent all possible sequences and columns represent different intervention phases. A = sugar-sweetened beverage; B = non-nutritive sweetened beverage; C = water.

**Table 3:** List of the Intervention Beverages and Types of NNS Sweeteners.

Group			
SSB Arm (42 g Sugars, 140 kcal)	NSB Arm (0 g Sugars, 0 kcal)	NNS Sweetener	Water Arm (0 g Sugars, 0 kcal)
Coca-Cola	Diet Coke	Aspartame and acesulfame–potassium	Still
	Coke Zero	Aspartame and acesulfame–potassium	
Pepsi	Diet Pepsi	Aspartame and acesulfame–potassium	Carbonated
Canada Dry Ginger Ale	Diet Canada Dry Ginger Ale	Aspartame and acesulfame–potassium	
Sprite	Sprite Zero	Aspartame and acesulfame–potassium	
7UP	Diet 7UP	Aspartame and acesulfame–potassium	
Orange Crush	Diet Orange Crush	Sucralose	

All beverages are given in 355 mL servings. Kcal = kilocalorie; NNS = non-nutritive sweetener; NSB = non-nutritive sweetened beverages; SSBs = sugar-sweetened beverages.

**Table 4:** Power (Sample Size) Calculation for Main Trial and Ectopic Fat Sub-Study.

Outcome	Mean Change ± SD	Correlation	N	N (Corrected for 20% Attrition)	Alpha	Power (1-Beta) (%)
Primary Outcome Family						
Weighed UniFrac Distance	0.04 ± 0.07 [43]	0.70	60	75	Largest *p*-value at alpha 0.0375 *; If failed test second *p*-value at alpha 0.025	98
Glucose iAUC (mmol/L/min)	44.81 ± 113.00 (20%) [88,89]	0.70	60	75		89
Secondary Outcome Family						
Waist Circumference (cm)	1.00 ± 7.17 [35,90]	0.70	60	75	0.05	35
Body Weight (kg)	1.00 ± 10.10 [35,90]	0.70	60	75	0.05	20
Fasting Plasma Glucose (mmol/L)	1.0 ± 2.20 [91]	0.70	60	75	0.05	99
2-Hour Plasma Glucose (mmol/L)	1.40 ± 1.40 [91]	0.70	60	75	0.05	99
Matsuda ISI_OGTT_	0.35 ± 1.26 [91]	0.70	60	75	0.05	81
Ectopic Fat Sub-Study						
IHCL (^1^H-MRS) (%)	5.00 ± 10.00 [92]	0.67	25	32	0.05	80

* If pass: alpha of 0.05 is recycled to the secondary outcome family. If fail: alpha of 0.0125 is recycled to the secondary outcome family. 1H-MRS scan = proton magnetic resonance spectroscopy; iAUC = incremental area under the curve; IHCL = intrahepatocellular lipid; Matsuda ISI_OGTT_ =Matsuda whole body insulin sensitivity index; SD = standard deviation.

**Table 5:** Baseline Characteristics for the Main Trial and Ectopic Fat Sub-Study.

Variable	Main Trial (N = 80)	Ectopic Fat Sub-Study (N = 32)
Anthropometry	Mean ± SD	Mean ± SD
Age (years)	42.34 ± 12.99	42.16 ± 12.91
Females, n (%)	41 (51)	16 (50)
Height (cm)	167.19 ± 10.66	167.90 ± 9.23
Weight (kg)	93.99 ± 18.89	95.25 ± 19.68
BMI (kg/m^2^)	33.71 ± 6.75	33.70 ± 6.03
Waist circumference (cm)	108.69 ± 13.50	110.31 ± 13.67
Systolic blood pressure (mmHg)	116.37 ± 12.49	76.68 ± 9.12
Diastolic blood pressure (mmHg)	76.24 ± 9.03	72.34 ± 9.76
FPG (mmol/L)	5.57 ± 1.19	5.77 ± 1.75
2 h-PG (mmol/L)	7.26 ± 3.11	7.99 ± 4.05
IHCL (%)	NA	9.7 ± 9.2
Self-reported ethnicity	n (%)	n (%)
Aboriginal	2 (3)	1 (3)
European	36 (45)	18 (56)
African/Caribbean	5 (6)	2 (6)
Latin American	6 (8)	3 (9)
Indian	5 (6)	2 (6)
East Asian	6 (8)	1 (3)
Southeast Asian	6 (8)	2 (6)
Mixed ethnicity	14 (18)	3 (9)
Highest level of education	n (%)	n (%)
Grade 9	1 (1)	0 (0)
High School Diploma or High School Equivalent	18 (23)	10 (31)
College Certificate or Diploma	18 (23)	9 (28)
Undergraduate Degree	27 (34)	8 (25)
Graduate Degree (including post-graduate)	16 (20)	5 (16)
Work status	n (%)	n (%)
Full-time (≥32 h/week)	40 (50)	14 (44)
Part-time (≤32 h/week)	13 (16)	5 (16)
Casual employee	6 (8)	6 (19)
Stay at home parent	6 (8)	2 (6)
Full-time student	3 (4)	1 (3)
Disability	3 (4)	1 (3)
Multiple work status	6 (8)	2 (6)
Other	3 (4)	1 (3)
Alcohol intake	n (%)	n (%)
None	21 (26)	6 (19)
1–2 times per year	9 (11)	4 (13)
Every 2–3 months	16 (20)	9 (28)
1–2 times per month	16 (20)	3 (9)
1–2 times per week	15 (19)	8 (25)
Daily	3 (4)	2 (6)
Regular medication use	n (%)	n (%)
Participants taking medications	23 (29)	12 (38)
Aspirin	1 (4)	1 (8)
Paracetamol	1 (4)	0 (0)
Combined inhaled corticosteroids and short-acting bronchodilators	1 (4)	0 (0)
Oral contraceptive	2 (9)	1 (8)
Statins	1 (4)	0 (0)
Antihistamine	2 (9)	0 (0)
Anxiolytic/anticonvulsant	1 (4)	0 (0)
Migraine relief	1 (4)	1 (8)
PReP	1 (4)	1 (8)
Short-acting bronchodilators	1 (4)	0 (0)
Stimulant	1 (4)	1 (8)
Topical corticosteroid	1 (4)	1 (8)
Mixed	9 (39)	6 (50)
ACEi + statin	1 (11)	1 (17)
Stimulant + diuretic + ACEi + antidepressant + antipsychotic + anxiolytic/anticonvulsant + medical marijuana + combined inhaled corticosteroids and long-acting bronchodilators + antacid	1 (11)	0 (0)
Short-acting bronchodilator + combined inhaled corticosteroids and long-acting bronchodilators + inhaled corticosteroid	1 (11)	1 (17)
Short-acting bronchodilators + antihistamine	1 (11)	1 (17)
Aspirin + Feminizing hormone therapy + oral disinfectant and antiseptic + topical antibiotics + diuretic	1 (11)	1 (17)
Combined ARB and diuretic +statin	1 (11)	0 (0)
ARB + Ursodiol	1 (11)	1 (17)
Statin + Aspirin	1 (11)	0 (0)
Antihistamine + ibuprofen	1 (11)	1 (17)
Supplement use	n (%)	n (%)
Participants taking supplements	23 (29)	8 (25)
Recreational marijuana	2 (9)	1 (13)
Multivitamin	6 (26)	3 (38)
Vitamin B	1 (4)	1 (13)
Vitamin C	2 (9)	0 (0)
Calcium	1 (4)	0 (0)
Iron	1 (4)	0 (0)
Fiber	1 (4)	0 (0)
Glucosamine	1 (4)	
Mixed	8 (35)	3 (38)
Multivitamin + fiber	1 (13)	1 (33)
Omega-3 + super cod liver oil + vitamin D + combination calcium and magnesium	1 (13)	0 (0)
Vitamin B + multivitamin	1 (13)	0 (0)
Omega-3 + turmeric	1 (13)	0 (0)
Zinc + vitamin B + vitamin C + fiber	1 (13)	1 (33)
Combination calcium and vitamin D + vitamin A + vitamin B + vitamin C + vitamin D + vitamin E + combination vitamin K and vitamin D + combination omega-3 and omega-6 + magnesium	1 (13)	0 (0)
Glucosamine + vitamin D + coEQ + combination omega-3 and vitamin E + ginkgo biloba + turmeric + multivitamin	1 (13)	0 (0)
Vitamin D + vitamin E + vitamin C	1 (13)	1 (33)
*Baseline background NNS intake*	*n (%)*	*n (%)*
From beverages	21 (26)	10 (31)
From foods	4 (5)	0 (0)
As added sweeteners	5 (6)	1 (3)
Multiple sources	6 (8)	1 (3)
None	44 (55)	20 (63)
Baseline SSB intake	n (%)	n (%)
Cola		
Coca-Cola	35 (44)	17 (53)
Pepsi	11 (14)	4 (13)
Non-cola		
Canada Dry Ginger Ale	21 (26)	6 (19)
Sprite	7 (9)	4 (13)
7-UP	3 (4)	0 (0)
Orange Crush	3 (4)	1 (3)
Baseline mean SSB intake/day	Mean servings/day * (range)	Mean servings/day * (range)
Overall	1.84 (1–5)	2.02 (1–5)
Cola		
Coca-Cola	1.93 (1–5)	2.00 (1–5)
Pepsi	2.27 (1–4)	3.00 (2–4)
Non-cola		
Canada Dry Ginger Ale	1.53 (1–5)	1.94 (1–5)
Sprite	1.86 (1–4)	1.50 (1–2)
7-UP	2.00	0.00
Orange Crush	1.00	1.00
NSB equivalents (NNS blends)	n (%)	n (%)
Cola		
Coke Zero (Asp and Ace-K)	18 (23)	9 (28)
Diet Coke (Asp and Ace-K)	15 (19)	7 (22)
Diet Pepsi (Asp and Ace-K)	11 (14)	4 (13)
Diet Coke or Coke Zero (Asp and Ace-K)	2 (3)	1 (3)
Non-cola		
Canada Dry Diet Ginger Ale (Asp and Ace-K)	20 (25)	5 (16)
Sprite Zero (Asp and Ace-K)	7 (9)	4 (13)
Diet Orange Crush (Sucralose)	4 (5)	2 (6)
Diet 7-UP (Asp and Ace-K)	3 (4)	0 (0)

^1^H-MRS = proton magnetic resonance spectroscopy; 2 h-PG = 2-h postprandial glucose; ACEi = angiotensin-converting enzyme inhibitor; Ace-K = acesulfame potassium; ADD = attention deficit disorder; ADHD = attention deficit hyperactive disorder; ARB = angiotensin II receptor blocker; Asp = aspartame; BMI = body mass index; CoEQ = co-enzyme Q10; ICHL = intrahepatocellular lipid; FPG = fasting plasma glucose; NA = not applicable; NNS= non-nutritive sweetener; NSB = non-nutritive sweetened beverage; PPI = proton-pump inhibitor; PReP = pre-exposure prophylaxis; SD = standard deviation; SSB = sugar sweetened beverage. * One serving was defined as 355 mL.

## Data Availability

Not applicable.

## Supplementary Materials

The following supporting information can be downloaded at: https://www.mdpi.com/2072-6643/15/5/1238/s1, File S1: STOP Sugars NOW Study Protocol.
