# Supplementary material for: Rationale, Design and Participants Baseline Characteristics of a Crossover Randomized Controlled Trial of the Effect of Replacing SSBs with NSBs versus Water on Glucose Tolerance, Gut Microbiome and Cardiometabolic Risk in Overweight or Obese Adult SSB Consumer: Strategies to Oppose SUGARS with Non-Nutritive Sweeteners or Water (STOP Sugars NOW) Trial and Ectopic Fat Sub-Study
Source: Nutrients. 2023 Feb 28;15(5):1238. doi: 10.3390/nu15051238 (PMC10005063; doi:10.3390/nu15051238)
Supplement: Supplementary file 1 [file nutrients-15-01238-s001.zip › nutrients-2209141-supplementary.pdf]

nutrients

MDPI

Supplementary Materials

Rationale, Design and Participants Baseline Characteristics of a Crossover Randomized Controlled Trial of the Effect of Replacing SSBs with NSBs versus Water on Glucose Tolerance, Gut Microbiome and Cardiometabolic Risk in Overweight or Obese Adult SSB Consumer: Strategies to Oppose SUGARS with Non-Nutritive Sweeteners or Water (STOP Sugars NOW) Trial and Ectopic Fat Sub-Study

Sabrina Ayoub-Charette [1,2], Néma D. McGlynn [1,2], Danielle Lee [1,2], Tauseef Ahmad Khan [1,2], Sonia Blanco Mejia [1,2], Laura Chiavaroli [1,2], Meaghan E. Kavanagh [1,2], Maxine Seider [3], Amel Taibi [1], Chuck T. Chen [1], Amna Ahmed [1,2], Rachel Asbury [4,5], Madeline Erlich [1,2,5], Yue-Tong Chen [1], Vasanti S. Malik [1,6], Richard P. Bazinet [1], D. Dan Ramdath [7,8], Caomhan Logue [9], Anthony J. Hanley [1,10,11], Cyril W. C. Kendall [1,2,8], Lawrence A. Leiter [1,2,12,13,14], Elena M. Comelli [1] and John L. Sievenpiper [1,2,12,13,14,*]

1. Study Title

A randomized controlled trial of the effect of replacing sugar-sweetened beverages with non-nutritive sweetened beverages or water on gut microbiome and metabolic outcomes: Strategies To OPpose SUGARS with Non-nutritive sweeteners Or Water trial

2. Running Title: Stop Sugars Now Trial

3. Funding: Canadian Institutes for Health Research (CIHR)

4. Principal Investigator

Prof. John L Sievenpiper, MD, PhD, FRCPC
Associate Professor, Department of Nutritional Sciences, University of Toronto
Staff Physician, Division of Endocrinology & Metabolism, St. Michael's Hospital
Scientist, Li Ka Shing Knowledge Institute, St. Michael's Hospital
St. Michael's Hospital
#6137-61 Queen Street East, Toronto, ON, M5C 2T2, CANADA
Telephone: 416-867-3732
Mobile: 416-305-4288
Fax: 416-867-7495
Email: john.sievenpiper@utoronto.ca

5. Co-Investigators

Lawrence A. Leiter, MDCM, FRCPC
Division of Endocrinology and Metabolism
St. Michael's Hospital
#6121-61 Queen Street East, Toronto ON, M5C 2T2, Canada

Telephone: 416-867-7441
Email: leiterl@smh.ca

6. Post-doctoral Fellow

Tauseef Khan, PhD
St. Michael's Hospital
61 Queen Street East
Toronto, ON, M5C 2T2, CANADA
Email: tauseef.khan@utoronto.ca

7. Graduate Students

Néma McGlynn, RD
St. Michael's Hospital
61 Queen Street East
Toronto, ON, M5C 2T2, CANADA
Email: nema.mcglynn@mail.utoronto.ca

Sabrina Ayoub-Charette
St. Michael's Hospital
61 Queen Street East
Toronto, ON, M5C 2T2, CANADA
Email: sabrina.ayoubcharette@mail.utoronto.ca

8. Background and Significance

International health agencies [1] and chronic disease associations [2,3] have called for reductions in free/added sugars to ≤ 5–10% of energy to address the growing epidemics of obesity and diabetes. Attention has focused especially on the reduction of the major source of free sugars, sugar sweetened beverages (SSBs), the excess consumption of which has been associated with weight gain, diabetes, and their downstream complications including hypertension, and coronary heart disease (CHD) [4–7]. Ontario's Healthy Kids Panel [8], Health Canada [9,10], the Standing Senate Committee [11], the Heart and Stroke Foundation [2] and Diabetes Canada [3] have recommended policies to reduce SSBs including replacement strategies, taxation, and/or bans on advertising to children. A role for non-nutritive sweeteners (NNSs) in these policy options has been conspicuously absent.

There is an emerging concern that NNSs may contribute to an increase in the diseases that they are trying to prevent. Systematic reviews and meta-analyses of prospective cohort studies have shown that NNSs are associated with increased risk of weight gain, diabetes, and CHD [5,12]. Although this evidence is recognized to be at high risk of reverse causality and disagrees with the higher quality evidence from randomized controlled trials [13], several biological mechanisms have been proposed, among them changes in gut microbiome. One highly-influential study [14] concluded that NNSs induce glucose intolerance through a loss of diversity in microbiome. This study, however, disagreed with a subsequent study [15] and had several methodological weaknesses including the lack of a control group. Despite the uncertainties, these data have contributed to a negative view of NNSs in the media [16–23].

There is an urgent need to address the ongoing concerns related to NNSs. Health Canada, in particular, has indicated that studies of sugar reduction strategies that use NNSs and target microbiome are an important research priority [24]. We propose to conduct a CIHR-funded randomized controlled trial that assesses the effect of a 'real world' strategy to reduce SSBs using non-nutritive sweetened beverages (NSBs) or water on gut microbiome, glucose tolerance, and cardiometabolic risk factors in overweight or obese participants.

9. Objectives

1. To assess the effect of replacing SSBs with NSBs or water on one primary outcome of diversity of gut microbiome over 4-weeks in overweight or obese participants.
2. To assess the effect of replacing SSBs with NSBs or water on another primary outcome of glucose tolerance over 4-weeks in overweight or obese participants.

3. To assess the effect of replacing SSBs with NSBs or water on the secondary and exploratory outcomes of body weight, blood pressure, glucose and insulin regulation, blood lipids, ectopic fat, liver fat, body adiposity, and diet quality over 4-weeks in overweight or obese participants

10. Hypothesis

1. Replacing SSBs with NSBs or water will change the diversity of gut microbiome after four weeks in regular SSB drinkers.
2. Replacing SSBs with NSBs or water will increase glucose tolerance after four weeks in regular SSB drinkers.
3. Replacing SSBs with NSBs or water will decrease body weight, reduce blood pressure, improve glucose and insulin regulation, decrease blood lipids, decrease ectopic fat, decrease liver fat, decrease body adiposity, and improve diet quality after four weeks in regular SSB drinkers.

11. Methods

11.1. Trial Design.

We will conduct a single-centre, open label, randomized controlled crossover trial with three four-week intervention phases (SSB, NSB, water) comparing the effect of replacing SSBs with NSBs or water on the gut microbiome and glucose tolerance. A cross-over design was selected due to the high inter-individual heterogeneity in gut microbiome [25]. Prior to randomization, participants will undergo a ≥ 2-week run-in phase to gather data on SSB consumption prior to study start. Each participant will then act as their own control receiving the 3 interventions in random order, each separated by a ≥ 4-week wash-out phase (**Figure S1**). We will collect fecal samples from participants and conduct Oral Glucose Tolerance Tests (OGTTs) before the start and at the end of each intervention for gut microbiome analysis and glucose tolerance. All study visits will occur in Toronto at the Clinical Nutrition and Risk Factor Modification Centre at St. Michael's Hospital. The microbiome analysis and the objective biomarkers of SSBs assays will be performed at the Department of Nutritional Sciences, University of Toronto; plasma glucose analysis will be performed at the Banting and Best Diabetes Center (BBDC), University of Toronto; plasma insulin analysis and other blood analyses (for a selected sample using a multiplex kit for adiponectin, adipsin, C-Peptide, Ghrelin, GIP, GLP-1, Glucagon, Insulin, leptin, PAI-1, Resistin and Visfatin) will be conducted at the Guelph Research and Development Centre, Ontario, Canada. Lipid analyses will be performed at St. Michael's Hospital Core Lab.

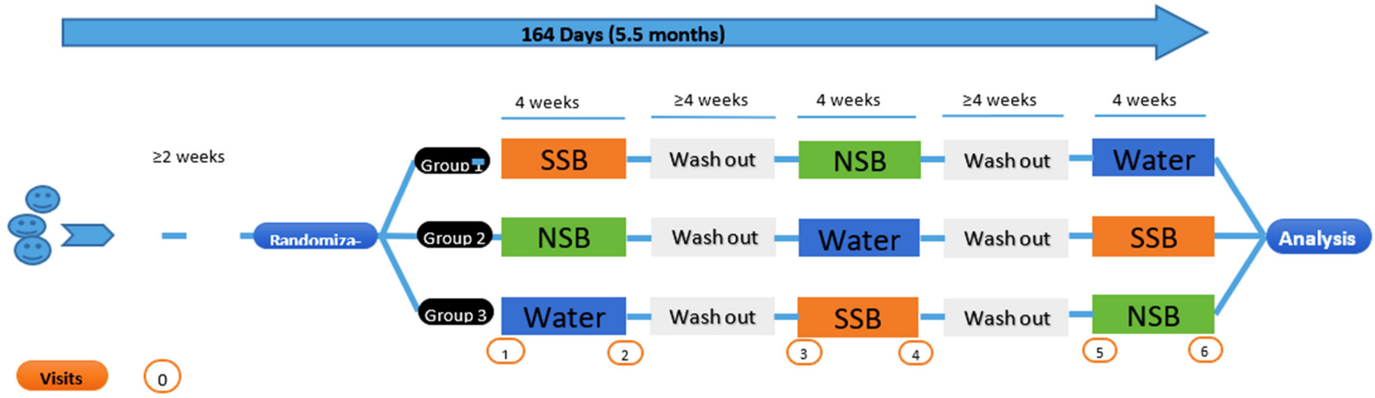

Figure S1. Overview of the study visits and phase *. *All possible groups are not shown.

11.2. Blinding

Blinding of the participants and investigators will not be possible due to packaging, taste and look of the interventions. However, outcome assessors (laboratory, microbiome analysis) and the statistician will be blinded to the identity of the treatments.

11.3. Participants

We will recruit 81 overweight or obese (ethnic specific cut-offs for overweight and obesity are used ≥ 23 kg/m² for Asian individuals and ≥ 25 kg/m² other individuals[11]), adult (age, 18–75 years) men and non-pregnant women (with no conception plans for the study duration) with a high waist circumference (≥ 94cm in men, ≥ 80cm in women in Europid, Sub-Saharan African, Eastern Mediterranean, and Middle Eastern individuals; ≥ 90cm in men and ≥ 80 cm in women for South Asian, Chinese, Japanese, and South and Central American individuals [26]) who are otherwise healthy (free of disease or illness), not taking medications that have a clinically relevant effect on the primary outcomes, not smoking, have no intentions of making major alterations to their diet or physical activity regime during study duration, and consuming ≥ 1 servings of SSBs per day (for which there is an NSB equivalent that contains the non-nutritive sweeteners acesulfame potassium or sucralose). Participants must be agreeable to consume only one type of SSB and brand-matched NSB for each month of intervention. If participants are consuming NSBs in addition to SSBs they must be willing to forgo them for the run-in and duration of the study. All participants must have a primary care physician and be proficient in English to be eligible for the study. Please see **Table 1** for a full set of exclusion criteria. SSBs will be defined as any sugar-sweetened beverage (sodas and soft drinks) that contains at least 50 kcal per 8-oz serving. For the purpose of the study, SSBs will NOT include fruit drinks, sports/energy drinks, sweetened iced tea, coffee, or homemade SSBs such as frescas or 100% fruit juice. All participants will be expected to participate in the study for approximately six months (one run-in phase of two weeks, three intervention phases of four weeks each, and two wash-out phases of four weeks each) and be able to attend all study visits. They must consent to have the study drinks shipped to their home address by a third-party company, who will not be informed of their participation in the study, or be agreeable to pick up study drinks from the study site at St. Michael's Hospital.

Of the 81 participants, 30 of them will be asked to consent to have an MRI taken to measure their liver and muscle adiposity.

Table S1. Study exclusion criteria*.

1. Age < 18 or > 75 years
2. BMI < 23 kg/m² for Asian individuals and < 25 kg/m² other individuals
3. Waist circumference < 94 cm in men, < 80 cm in women in Europid, Sub-Saharan African, Eastern Mediterranean, and Middle Eastern individuals; < 90 cm in men and < 80 cm in women for South Asian, Chinese, Japanese, and South and Central American individuals
4. Not regularly drinking SSBs (≥ 1 serving per day)
5. Self-reported pregnant or breast feeding females, or women planning on becoming pregnant throughout study duration Regular medication use that have a clinically relevant effect on the primary outcomes (except birth control and PRN meds such as Advil, Tylenol, etc)
6. Antibiotic use in the last 3 months
7. Complementary or alternative medicine (CAM) use as deemed inappropriate by investigators
8. Self-reported diabetes
9. Self-reported uncontrolled hypertension (or systolic blood pressure (BP) ≥ 160 mmHg or diastolic BP ≥ 100 mmHg [69])
10. Self-reported polycystic ovarian syndrome
11. Self-reported cardiovascular disease
12. Self-reported gastrointestinal disease
13. Previous bariatric surgery

14. Self-reported liver disease
15. Self-reported uncontrolled hyperthyroidism or hypothyroidism
16. Self-reported kidney disease
17. Self-reported chronic infection
18. Self-reported lung disease
19. Self-reported cancer/malignancy
20. Self-reported schizophrenia spectrum and other psychotic disorders, bipolar and related disorders, and dissociative disorders
21. Major surgery in the last 6 months
22. Other major illness or health-related incidence within the last 6 months
23. Smoker
24. Regular recreational drug users
25. Heavy alcohol use (> 3 drinks/day)
26. Do not have a primary care physician
27. Participation in any trials within the last 6 months or for the duration of this study
28. Any condition or circumstance which would prevent you from having an MRI (e.g., having prostheses or metal implants, tattoos, or claustrophobia) (this is applicable for the 30 participants in the MRI portion of the study)
29. Individuals planning on making dietary or physical activity changes throughout study duration
30.

Copyright: © 2023 by the authors. Licensee MDPI, Basel, Switzerland. This article is an open access article distributed under the terms and conditions of the Creative Commons Attribution (CC BY) license (https://creativecommons.org/licenses/by/4.0/).

* Disease exclusions will be based upon self-reported diagnosis.

11.4. Sample Size (Power) Calculation.

The study will be performed in a total of 81 participants. It is powered to show a difference between the water, NSB, and SSB arms in 60 participants in the two primary outcomes. Assuming a drop-out rate of 20 percent, we would need 75 participants in order to have to power to detect a difference. Since our final recruitment strategy resulted in a large number of participants who made it through the run-in phase, we decided to increase our recruitment in order to replace drop outs and thus will recruit 81 participants.

One primary outcome is in beta diversity of the gut microbiome communities of the participants between water and NSB groups via 16S ribosomal rRNA gene sequencing. We used the micropower R package to compute sample size based upon the power of 16S tag sequencing that can be analyzed using pairwise weighted UniFrac distances [28]. UniFrac is a distance metric based upon the unique fraction of branch length in a phylogenetic tree built from two sets of taxa. Comparison of microbiome samples is performed via weighted UniFrac, which considers the relative abundance of taxa. We simulated the within-group distance as 0.2, and the standard deviation (SD) of within-group distances as 0.07. To detect a weighted UniFrac distance of 0.04, which is smaller than the effect observed in a studies of Suez et al. [29] (0.05 derived from Figure 5), and considering it is a cross-over study with a within-person correlation of 0.7, and taking into account multiple arms we calculated that for above 95 percent power we would need 60 participants in this study. Assuming a loss of 20 percent, we will recruit 75 participants. We are confident about detecting an alteration in

gut microbiome diversity if it exists as previous studies show that small changes in diet causes significant alteration in gut microbiome taxa over a much shorter period (5 to 7 days) in fewer individuals (10 to 25 people) [25,30].

Another primary outcome is glucose tolerance, as measured by incremental Area Under the Curve (iAUC) from a 2-hour 75 g OGTT. With 60 participants we will have 89 percent power (assuming absolute numbers for mean and SD from our recent unpublished randomized trial) to detect a 20% change in mean iAUC between the water and NSB group if the direction of change is similar to Suez et al. [124] while assuming a within-person correlation of 0.7 and taking into account the three comparisons. The 20% difference for glucose iAUC is based on the minimally important difference proposed by Health Canada to support postprandial blood glucose response reduction claims [31].

This power calculation takes into account adjustment for multiple testing for both primary outcomes using the Benjamini-Hochberg procedure, which is a suggested method by the Food and Drug Administration in its "Multiple Endpoints in Clinical Trials Guidance for Industry" [32]. Benjamini-Hochberg procedure is a step-down method that controls for false discovery rate, while maintaining high power [33,34]. We will implement a truncated Benjamini-Hochberg method with parallel gatekeeping in which some portion of the unused alpha from each step is reserved for passing to the secondary outcome family if any of the primary outcome is significant. The alpha levels calculated for the primary outcomes is given in **Table S2**. The study is also powered to show a difference between the three arms for secondary outcomes with an $\alpha = 0.0125$, the lowest possible starting α for secondary outcomes based upon the truncated Benjamini-Hochberg procedure.

Sub-study: 1H-MRS will be performed in 25 subjects to assess intrahepatic and intramuscular fat. We will have 99% to show a difference in liver fat of 5% between the water and NSB arm for both hepatic and muscle fat assuming a between group SD of 4%, with a correlation of 0.65 and alpha of 0.05.

Table S2. Power calculation for primary outcomes between water and NSB arms.

Outcome type	Outcome	Mean change	SD	Correlation	N	Alpha*	Power (Beta)	N (corrected for 20% loss to follow up)
**Primary outcomes family**								
**Primary**	Microbiome UniFrac Distance	0.04	0.07	0.7	60	0.0375	98%	75
**Primary**	Glucose iAUC	44.81 mmol/L/min (20%)	113 mmol/L/min	0.7	60	0.025	89%	75

P-value order will be from largest to smallest.

11.5. Recruitment

Participants will be recruited through postings and flyer handouts at St. Michael's Hospital, the University of Toronto, local clinics, and surrounding areas, advertisements in newspapers and on transit, and online listings on Craigslist and Kijiji. Some participants will complete a Survey Monkey to assess general eligibility. We will be utilizing a digital marketing group, TrialFacts, to launch a campaign to recruit participants. TrialFacts works exclusively with research groups to provide specialized patient recruitment services for clinical trials. Participants will be screened first by telephone. Eligible participants will be called in for an information session on the study including being provided the consent form. Those that wish to participate will provide written consent and will subsequently have an in-person screening including blood work. Based on our most recent FACE trial (NCT02459834), we estimate a recruitment rate of 1–2 participants/week and therefore plan to recruit all the participants within a 6-month period. **Dr. Tauseef Khan** will organize the recruitment. Our **MSc candidate #1 (Néma McGlynn, RD)** and our **Research Coordinator** will conduct the screening.

11.6. Screening and Consent

Interested individuals will call the study coordinator and be screened over the phone for eligibility using a questionnaire and be briefed on the nature of the study, treatments, and procedures. Individuals who qualify and are interested will be asked to come to the Clinical Nutrition and Risk Factor Modification Centre at the St. Michael's Hospital (study site) for an information session where they will be given the consent form. They will be permitted to take

the form home so that ample time to review study procedures. Participants who provide written consent will be assigned a screening identification (ID) and will be scheduled for an in-person screening will be arrange to obtain anthropometric measures as well as questionnaire administration regarding SSB intake to confirm study qualification. The anthropometrics will include height, weight, waist circumference, and blood pressure. Automated Office Blood Pressure (AOBP) measurements at the first screening visit will be recorded in both arms unless a physical condition necessitates the use of a particular arm. The arm with the highest mean systolic measurements at screening will then be used for all subsequent AOBP assessment throughout the study. If the mean of 3 AOBP measurements in the arm with the highest systolic measurements shows systolic blood pressure (SBP) ≥ 160 mmHg or diastolic BP (DBP) ≥ 100 mmHg, then the patient will be excluded for uncontrolled hypertension [27].

Any significant results will be provided to participant's family physician for further follow-up. Those who meet all the eligibility criteria will be enrolled in the run-in phase of the study, which will last two weeks, and then be assigned a study ID. Participants will be instructed on details of the trial protocol.

11.7. Run-in Phase

Participants who are eligible following in-person screening will also start the ≥ 2-week run-in phase. For two weeks during the run-in phase and prior to the first study visit, participants will complete a beverage log sheet. They will also be instructed on how to collect a 24-hour urine sample and fecal sample and provided kits to do so for the first study visit. The participants will be provided with their beverage log sheets for the month intervention. During the run-in phase participants will begin to log their usual beverage intake as well as asked for their beverage preference of SSB and NSB.

11.8. Randomization

Using the Research Electronic Data Capture (REDCap) program, the Applied Health Research Centre (AHRC) will perform the randomization with no stratification. Following successful completion of the run-in phase and following measurements taken at the first study visit, participants will be randomized into groups, of a possible six, using a blocked (Latin squares) randomization. These groups will be sequences representing SSB, NSB and water groups. The Latin square sequences will be randomly allocated to participants with a similar number of participants allocated to each treatment sequence (**Table 2**). The randomization schedule is also created by AHRC through REDCap. The participants will only be randomized and given their study drinks once all measures from the first study visit are collected.

Table 2. Latin square randomization for three group cross-over study.

	Intervention Phase 1	Intervention Phase 2	Intervention Phase 3
Sequence Group 1	A	B	C
Sequence Group 2	B	C	A
Sequence Group 3	C	A	B
Sequence Group 4	B	A	C
Sequence Group 5	A	C	B
Sequence Group 6	C	B	A

Rows represent all possible sequences and columns represent different intervention phase.

11.9. Intervention

There will be three interventions: Participants will be provided with the SSB of their choice from **Table S3** (355 mL, 140 kcal, 42 g sugars per can), equivalent NSB (355 mL, 0 kcal, 0 g sugars per can), or water (355 mL, 0 kcal, 0 g sugars per can or bottle of still or carbonated water) to replace the amount of SSBs they usually consume (≥ 1 serving/day) as determined in the run-in phase. All intervention beverages will be provided to each participant. They will be instructed to replace their usual SSBs with the study beverages while freely consuming their usual background diets. The calories of the intervention groups will not be matched to allow for "real-world" substitutions using products available on the market. They will pick up one week of their beverage assignment at the first visit of each phase and then will have the remaining three weeks of beverages delivered using an online grocery delivery service (i.e., Grocery

Gateway (www.grocerygateway.com)). Our **MSc candidate #1 (Néma McGlynn, RD)** and our research coordinator will coordinate the beverage disbursements. The participants will receive relevant drinks during the intervention phase based on their group assignment. They will revert to their usual SSB intake during wash-out phase during which they will not receive any beverages from the study.

Table S3. A full list of the three interventions and available beverages is given below.

SSBs group – 355 ml can 42 grams of sugars	NSBs group – 355 ml can 0 grams of sugar	Water group – 355 ml bottle or can
Coca-Cola	Diet Coke (Aspartame, Acesulfame)	
	Coca Cola Zero (Aspartame, Acesulfame)	
Pepsi	Diet Pepsi (Aspartame, Acesulfame)	
Canada Dry Ginger Ale	Diet Canada Dry Ginger Ale (Aspartame, Acesulfame)	
Schweppes Ginger Ale	Diet Schweppes Ginger Ale (Acesulfame, Sucralose)	
Mountain Dew	Diet Mountain Dew (Aspartame, Acesulfame, Sucralose)	
Sprite	Sprite Zero (Aspartame, Acesulfame)	
7UP	Diet 7UP (Aspartame, Acesulfame)	
Orange Crush	Diet Orange Crush (Sucralose)	

11.10. Study visits (Week-0 and Week-4 for all 3 interventions, 6 total study visits):

The trial will be conducted at the CFI-funded Toronto 3D Clinical Research Centre and MRI Research Unit and at the Clinical Nutrition and Risk Factor Modification Centre at St. Michael's Hospital. Each participant will receive the three interventions in random order, each separated by a ≥ 4-week washout. Participants will receive motivational phone calls every 2-weeks between visits to provide reminders and ensure fidelity to the protocol.

For Week-0 of first intervention only:

The first visit will be scheduled upon finalizing the run-in phase. Participants will come to the study site and their beverage logs will be checked for completion for the 2-week run-in phase, any changes required will be communicated. Participants will be notified of their randomization assignments after completing all of their baseline testing.

For all six study visits:

Participants will come to the study site prior to their visit to pick up standard stool and urine collection kits as well as be instructed on how to complete a three-day diet record (3DDR) using Nutritics, a nutrition software available for use on smartphones (a paper form of the 3DDR will be available for participants who do not have access to a smartphone or prefer not to use the application). Participants will be provided with instructions for stool collection, storage, and delivery to the study centre. Stool samples will be collected in specimen containers surrounded by dry ice packs, and brought to the centre by the participants and stored in a −80C freezer. At each visit, study staff will retrieve the stool sample, the 24-hour urine collection, and beverage logs. Subjects will come in to the study site in a 10 to 12-hour fasted state. They will be instructed to maintain the same dietary and exercise patterns the evening before each test and consume a minimum of 150 g of carbohydrate each day over the three days prior to the test. To ensure that these instructions are followed, participants will be provided with examples of what constitutes 150 g of carbohydrate. Their weight, waist circumference, and blood pressure will be measures. A fasting blood sample for primary, secondary, and exploratory outcomes (including glucose, insulin, lipid panel, ALT and other liver enzymes, and other markers of glucose control, biomarkers of adherence, and other biomarkers of cardiometabolic risk) will be taken by an intravenous nurse. A standard protocol will be followed for the administration of a 2-hour 75 g-OGTT. Participants will have a catheter inserted into a forearm vein by the nurse, secured by tape, and kept patent by saline. Two baseline samples will be obtained in the fasting state at 0-min. A 75 g OGTT meal (GlucoDex 75 g, Rougier Pharma Mirabel, QC, Canada) will then be administered with instructions to consume it over exactly 5 min at a constant rate using a timer. Additional

blood samples will be drawn at 30, 60, 90, and 120 min after the start of the test, at the end of the test the nurse will remove the catheter. Breakfast will be prepared at the study site kitchen and provided to the participants after the OGTT. Staff will also administer case report forms and questionnaires which will include information on diet (cravings, hunger and fullness), physical activity, medication changes, and symptoms. The study dietitians will review the 3DDR with participants. For the 30 participants who will participate in the imaging portion of the study, a 1H-MRS scans will be conducted in the CFI-funded Toronto 3D Centre MRI research unit at St. Michael's Hospital. The 1H-MRS will be performed to measure ectopic fat in liver and calf muscles (**Table S4**). Once all measures have been collected, the participant will be provided the intervention. For week-0 of all 3 interventions, participants will be given 1 week's worth of beverages to take home; the beverages for the remaining 3 weeks will be delivered using an online delivery service (Starhawk express (https://www.starhawkexpress.com/)). They will also be provided with their beverage log sheets for the month

Washout (2 x 4 weeks):

At the end of week-4 for the first two interventions, the wash-out phase of ≥ 4 weeks will start for which participants will be asked to revert back to their usual beverage intake. Participants will be given a beverage log to complete over this four-week phase. No beverages will be provided by the study site during the wash-out phase. The study coordinator will call the participants near the middle and near the end of each phase to remind them of their group assignment and the next study visit. Reminders will also be sent via email.

Table S4. Participant Visit Schedule.

Phase (Length in weeks)	Screening (0)	Run-in (2 weeks)	Intervention phase 1 (4 weeks)		Wash-out (4 weeks)	Intervention phase 2 (4 weeks)		Wash-out (4 weeks)	Intervention phase 3 (4 weeks)	
**Study Visit**	**0**	**No visit**	**Week 0 (Start)**	**Week 4 (End)**	**No visit**	**Week 0 (Start)**	**Week 4 (End)**	**No visit**	**Week 0 (Start)**	**Week 4 (End)**
Written Informed Consent Provided	x									
**Anthropometric measures**										
Height	x									
Weight	x		x	x		x	x		x	x
Waist circumference	x		x	x		x	x		x	x
Blood pressure	x		x	x		x	x		x	x
**Biochemical measures**										
Fecal sample			x	x		x	x		x	x
24-hour urine collectio (biomarkers for compliance)			x	x		x	x		x	x
Blood sample drawn			x	x		x	x		x	x
OGTT			x	x		x	x		x	x
**Questionnaires**										
Personal information	x									
Demographic Data	x									
Medical History	x									
Case Report Form (eg. medications, physical activity)	x		x	x		x	x		x	x
Beverage log	x	x	x	x	x	x	x	x	x	x
Symptoms questionnaire			x	x		x	x		x	x
Three-day diet record			x	x			x			x
Cravings/hunger/satiety questionnaires			x	x		x	x		x	x
**Radiology**										
Liver fat (MRS)			x	x		x	x		x	x

11.11. Outcomes

The **two primary outcomes** are change in gut microbiome beta diversity, measured by 16S rRNA gene sequencing, and plasma glucose iAUC, measured by OGTT.

Secondary outcomes are change in waist circumference, body weight, fasting plasma glucose, 2 hrs plasma glucose [2h-PG], and the Matsuda whole body insulin sensitivity index [Matsuda ISI_{OGTT}] [35].

Exploratory outcomes are change in blood pressure; fasting plasma insulin; 75g OGTT derived indices (iAUC plasma insulin, maximum concentrations (Cmax) and time to maximum concentrations (Tmax) of plasma glucose and insulin, mean incremental plasma glucose and insulin, homeostatic model assessment of insulin resistance (HOMA IR) and the insulin secretion-sensitivity index-2 [ISSI-2]); cardiometabolic risk (lipid panel) ectopic fat (an early metabolic lesion) in liver (intra-hepatocellular lipid [IHCL]) and calf muscles (intra-myocellular lipid [IMCL]) by ^{1}H-MRS; fasting blood lipid profile; satiety, hunger, and food cravings (using the Control of Eating Questionnaire), and diet quality (by analysis of the 3DDRs) [36].

Adherence outcomes will be based on participant beverage logs, returned beverage containers, and objective biomarkers of SSBs (increased 13C/12C ratios in serum fatty acids, increased urinary fructose), water (decreased 13C/12C ratios in serum fatty acids, decreased urinary fructose), and NSBs (urinary acesulfame potassium, sucralose) intake.

11.12. Compliance assessment

To assess compliance, participant beverage logs will be checked at each visit. In addition, participants will return unused SSBs, NSBs, or water bottles to assess compliance. Urinary analysis will be obtained for biomarker analysis for compliance (**Biomarkers of Adherence**, page 9).

11.13. Analytical Techniques

Anthropometric analyses

Height will be measured with a wall-mounted stadiometer (Perspective Enterprises, Portage, MI, USA).

Body weight will be assessed by beam scale.

Waist circumference will be assessed using the Heart and Stroke Foundation methodology [37].

Blood pressure (BP) and **resting heart rate** will be measured. To collect this measure, participants will remain seated in a quiet, temperature-controlled room for at least 5 minutes to achieve resting heart rate and BP. Subsequently, BP will be measured oscillometrically using the OMRON Intellisense HEM-907 according to JNC VII criteria. BP will be measured in triplicate, with each measurement separated by one minute, and the average of the three measures will be taken [38].

Biochemical analyses

Fecal microbiome analyses

DNA will be extracted from fecal samples and compositional analysis will be done by next generation sequencing. Primers will be used to target the **appropriate** regions of the *16S rRNA* gene for pair-end sequencing using Illumina MiSeq [39]. Sequencing data will be analyzed to assign Operational Taxonomic Units (OTUs) to determine their abundance. Data for selected taxa will be confirmed by qPCR [40]. Alpha and beta diversity indexes will be calculated. The metagenome will be inferred from compositional data *in silico* [41]**.**

Plasma Analyses

Plasma samples for glucose and insulin will be separated by centrifuge and the plasma immediately frozen at −80 °C at the University of Toronto, in a locked room, in a locked building for analysis. The BBDC at the University of Toronto will perform analyses of the plasma glucose using the glucose oxidase method [137]. The Guelph Research and Development Centre will perform the analysis for the plasma insulin using enzyme-linked immunosorbent assay (ELISA), a solid phase two-site enzyme immunoassay method using Mercodia AB Insulin ELISA (Uppsala, Sweden) kits [42]. Plasma glucose and insulin curves will be plotted as the incremental change over time and iAUC will be calculated geometrically for each participant using the trapezoid method, ignoring areas below the fasting value [43]. J. Alick Little Lipid Research Laboratory at St. Michael's Hospital will perform analyses of lipids (enzymatic method). ALT will also be analyzed at St Michael's Hospital Core Lab. Remaining samples will be stored at −80 °C for future

analysis of metabolomics. The Matsuda ISI (Matsuda ISI_{OGTT}) will be calculated using the 75 g-OGTT derived plasma glucose (PG) and insulin (PI) valuess, according to the formula by Matsuda et al. [44]: 10,000 divided by the square root of ([FPGFPI] ([mean PG mean PI]), where PG is expressed in mg/dl (0.0551 mmol/L) and PI in U/ml (6 pmol/L). The early insulin secretion index ($\Delta PI_{30-0}/\Delta PG_{30-0}$) will be calculated as the change in PI from 0 minutes to 30 minutes divided by the change in PG over the same period [45].

Biomarkers of adherence

Biomarkers of adherence will be assessed at the Department of Nutritional Sciences, University of Toronto by a novel gas chromatography, compound specific isotope ratio mass spectrometry (GC-CSIRMS) method that detects 13C/12C ratios in serum fatty acids (e.g., palmitoleic acid) derived from the de novo lipogenesis (DNL) of added sugars from sugar cane (sucrose) or corn (high fructose corn syrup [HFCS]) will assess adherence to SSBs (increased) or water (decreased). A liquid chromatography with mass spectrometry using electrospray ionization (LC-ESI-MS/MS) will be used to assess urinary sucralose or acesulfame potassium (Ace K) [46]. Urinary fructose will be measured by gas chromatography mass spectrometry (GS-MS) [47].

Questionnaires

Dietary Assessment

Participants will be asked to fill out a weighed 3DDR using Nutritics, a nutrition software available for use on smartphones. The 3DDR will consist of two non-consecutive weekdays and one weekend day during the run-in phase and during the last week of each intervention. If participants do not have access to a smartphone, a paper form of the 3DDR will be provided. Participants will also be provided with scales to insure the accuracy of the record. Registered dietitians will instruct and review 3DDRs with participants. The 3DDRs will be used to evaluate changes in diet quality and will be analyzed by using Nutritics. Food cravings, hunger, and satiety will be assessed using modified Corby Martin's Questionnaires [48].

Behavior of beverage consumption

Behavior of beverage consumption will be assessed with a product consumption questionnaire. This will be done at the last visit for the participants that are still enrolled in the study and via telephone or email for the participants that have completed the study.

Radiology

Liver and calf muscle fat

We will use a 3T Siemens Magnetom Skyra scanner (Erlangen, Germany) with either an 18 channel body phased array coil or a dedicated peripheral phased array coil. Single voxel, 30 mm cubed spectroscopy will be acquired for IHCL and IMCL using a STEAM (Stimulated Echo Acquisition Mode) sequence and the following acquisition parameters: TR 3000 ms, multiple TE values 12 ms, 24 ms, 36 ms, 48 ms, 72 ms, mixing time (TM) 10 ms, 10 averages, 1024 data points and receiver bandwidth 1200 Hz for an acquisition time under one minute. Participants will be scanned for 30 minutes.

Our **Postdoctoral Fellow (Dr. Tauseef Khan, MBBS, PhD**) will coordinate all analyses and our **MSc candidate #2 (Sabrina Ayoub-Charette)** will conduct fecal microbiome analyses.

11.14. Study duration

Each participant will be enrolled for approximately 6 months (~164 days) and will undergo 1 × 2-week run-in phase, 3 × 4-week interventions and 2 × 4-week wash-out phases. All participants will undergo each intervention for four weeks, the order being randomly assigned. The entire study is expected to take approximately 4 years to complete, allowing for ethics and material/data transfer agreement approvals (6 months), rolling recruitment (6-months), run-in

phase, intervention (×3) and washout (×2) phases (total 6-months), microbiome/ plasma/ imaging/ biomarker analyses (9-months), data analyses (9-months), and final write-up (6-months).

11.15. Data Integrity/Management

All paper copies of data forms will be stored under a double lock system to ensure confidentiality (locked cabinet in a locked office). Only PI, co-investigators and assigned study personnel will have access to the cabinet. The participant digital database will be securely stored centrally at SMH. The data management system enables anonymized and secure information storage, retrieval and sharing and will be organized and supported by the Li Ka Shing Knowledge Institute IT team of St. Michael's Hospital. All data forms will be stored under a double lock system to ensure confidentiality. Only the PI, co-investigators and assigned trial personnel will have access to the cabinet. The Applied Health Research Centre (AHRC), St. Michael's Hospital, will be contracted to manage the digital database securely and ensure data integrity. Their data management system enables anonymization, secure information storage, retrieval, and sharing of data.

11.16. Personal Health Information

We will be collecting personal health information during this study. The personal health information will include name, phone number, email address, demographic information, health history, questionnaires and records, and research visit records. In order to keep participant data confidential, we will create a master linking log which will separate identifying data (name and contact information) from study data with a study ID. The master linking log will be kept in a password-protected file on a drive through St Michael's Hospital computer, only accessible to study staff and investigators. Any subject requesting access to their personal health information will be able to do so by requesting this through the study coordinator.

11.17. Subject Compensation

Once participant is randomized travel compensation will be provided. To compensate for lengthy time required for study visits, each participant will be compensated $80 for first 5 visits and $95 for final visit, totaling $495 for completing the trial. Participants will receive payment at the end of each phase (three times during the study). However, they will be informed that if they choose to withdraw early, they will be compensated for each visit that was completed. Transportation costs (i.e., parking or TTC tokens) will be reimbursed and breakfast will also be provided at each study visit. Any transportation costs incurred for additional visits for obtaining stool and urine collection kits will be reimbursed as well.

11.18. Anticipated Challenges

Recruitment challenges

We require individuals who are regular SSB drinkers. In fact, soft drinks are the second largest source of added sugars for adults in Canada [49] and the per capita intake of SSBs in Canada is 0.9 with estimated 20 percent drinking more than 1 SSB serving per day [50]. Even if half of this population drinks NSBs, there is estimated 10 percent adult population that drinks more than 1 SSB serving per day. In addition, we plan to recruit from overweight and obese individuals who are known to have a higher SSB intake [51].

Compliance challenges

In dietary studies, compliance is usually a challenge. We will address this by having participants keep beverage logs at all phases of the trial including run-in and washout phases. Participants will be able to select a brand of beverage they like in both NSB and SSB arms. These beverages will be provided to the participants from the study site for the first week of each phase and shipped via Starhawk express for the three subsequent weeks. In addition, adherence to treatment will be reinforced with regular phone calls and/or emails to the participants. Since the intervention consists of a wide range of easily available supermarket drinks (**Table S3**), we do not anticipated challenges in acceptance on basis of palatability, taste or volume.

Protocol compliance

Those who use antibiotics during an intervention phase will have to go through a 30 days washout period at the completion of their antibiotic course and restart that phase of intervention. If the antibiotic course is taken during the washout phase, a 30 days additional washout will be instituted.

12. Statistical Analysis

Data will be analyzed according to an intention to treat (ITT) principle using mixed models in STATA 14 (StataCorp, Texas, USA). Sensitivity analysis will be performed on the basis of complete data availability for primary endpoints. A separate sensitivity analysis will be performed on the basis of antibiotic use during the trial.

Primary outcomes

Repeated measures mixed effect models will be used to assess changes in the two primary outcomes i) beta diversity and ii) glucose iAUC between the groups. Pairwise comparisons between interventions will be performed using Tukey-Kramer adjustment or other appropriate statistics. For all primary outcomes effect modification by sex will be explored. We will use the truncated Benjamini-Hochberg false discovery rate controlling method with parallel gatekeeping procedure to correct for multiple comparisons for all primary outcomes [32].

Secondary outcomes

Repeated measures mixed effect models be used to assess changes in weight, waist circumference, fasting glucose, 2 hrs plasma glucose, and MATSUDA. Pairwise comparisons between interventions will be performed using Tukey-Kramer adjustment or other appropriate statistics. For all secondary outcomes effect modification by sex will be explored. We will use the truncated Benjamini-Hochberg false discovery rate controlling method with parallel gatekeeping procedure to correct for multiple comparisons for all secondary outcome comparisons [33] if at least one primary outcome reaches significance. If none of the primary outcomes reach significance, the secondary outcomes will be analyzed as exploratory variables with no adjustment for false discovery rate.

Exploratory and adherence outcomes

Repeated measures mixed effect models will be used to assess changes in all exploratory outcomes without controlling for false discovery rate. Pairwise comparisons between interventions will be performed using Tukey-Kramer adjustment or other appropriate statistics. Effect modification by sex will be explored.

Subgroup Analysis

A priori analysis will be conducted by age, sex, ethnicity, baseline BMI, baseline WC, baseline FPG, baseline 2hPG (OGTT), baseline iAUC, medication use, sweetener blend (intervention), beverage type (intervention), and background sweetener use.

13. Significance of work

Replacement of SSBs is an essential recommendation in all dietary guidance on limiting added sugars. However, the role of NSBs to replace SSBs is complicated by its purported adverse effect on the composition of the gut microbiome with associated development of adverse glucose tolerance. Our study will be the first to investigate this question in humans in a randomized controlled trial comparing SSBs, NSBs and water. It will clarify if NSBs are similar to water and will inform the recommendations for dietary guidelines on replacements of added sugars such as SSBs. The proposed project will aid in knowledge translation related to the health effects of NSBs as alternative to sugars, informing evidence-based guidelines and improving health outcomes by educating healthcare providers and patients, stimulating industry innovation, and guiding future research design.

14. Timeline

Milestone	Date

Grant awarded	Apr 2017
Ethics Approval	Jan 2018
MTA/DTA Approval	Feb 2018
Recruitment commenced	Feb 2018
First patient randomized	Mar 2018
Recruitment completed	Aug 2018
Last patient completed	Mar 2019
Laboratory analysis completed	Oct 2019
Data analyses completed	July 2020
Manuscript completed	Nov 2020
Manuscript submitted	Dec 2020
Manuscript accepted	Jan 2021

15. Budget

**Budget Item**	**Subtotal**
Personnel	$150,000.00
Microbiome	$30,000.00
Participants	$42,000.00
Laboratory	$70,000.00
Beverages	$3,000.00
Office Supplies	$2,500.00
Recruitment	$20,000.00
Knowledge Translation	$12,500.00
**Grand Total**	**$330,000.00**

16. Genetic Testing: This aspect of the study will be optional and is not part of the main objectives of the study

Objective: To determine whether there are any genetic differences between individuals in their response to study beverages.

Background: There is considerable interest in the scientific community to study how genes may influence response to diet. In terms of taste our diets tend to be bland containing lower salt and also less sugar. The genes controlling these tastes may therefore determine intake [52,53]. This is an example of why comprehensive genetic analysis, due to the implication of a growing number of genes of interest, would be helpful. We will, however limit our analyses to specific genes currently known to affect dietary response. Generally, the effect of genes on diet is very modest and usually requires significant numbers of people to detect the difference. It therefore has very limited clinical relevance. We may therefore pool data from across our future studies (typically 30–80 participants per study). A bio-bank will be established for the proper storage and management of these samples.

We hope to make our initial findings known to participants at the end of the study, especially if we discover specific groups for which certain types of dietary advice may prove advantageous. Genetic testing results are solely for use in this study and will not be shared with any third parties (i.e., insurance companies or the hospital).

Analyses: The DNA will be extracted from "buffy coat" of white blood cells obtained after plasma has been removed from citrated blood samples drawn at week 0 of phase I or any time point during the main study. Therefore, no additional blood samples will be required for this study.

The genes of interest will include those related to the effectiveness of dietary change on blood glucose, cholesterol and associated measurements including blood pressure, taste preference, and other related genes which may influence response to diet.

Incidental findings: *An incidental finding is a finding concerning an individual research participant that has potential health or reproductive importance and is discovered in the course of conducting research but is beyond the aims of the study* [54]. Any incidental findings (as defined above) obtained from screening or during the course of the main study from laboratory tests, ultrasound or MRI images will be provided to the family physician for further investigation. The participant will be notified in reassuring terms and advised to follow-up with their family physician. In this sub-study, if genes identified have clinical implications the advice of experts in that particular field will be sort to arrange appropriate counseling for the participant. The family physician's involvement would be sought if the participant consents to it. However, the chances of incidental findings are limited since only specific genes known to affect dietary response will be studied.

Banking of Samples: Blood samples (de-identified) will be stored at the University of Toronto (at the Medical Science Building or the Fitzgerald Building) until all planned analyses have been undertaken-usually about 3 to 5 years. Optional longer-term banking will be made available to all participants for storage periods of 30 years after study completion to allow for use in future secondary and exploratory analyses in relation to the objectives of the current research project. At the end of each storage period, samples will be destroyed by autoclaving.

We are currently investigating the process of setting up a Biobank to efficiently and securely store and manage these samples; the necessary application will be sent to the St. Michael's REB once the project proposal is drawn up. Presently the use and ultimate destruction of these samples will be the responsibility of the investigator, Dr. Sievenpiper. However, detailed information on the organization and management will be in the biobank proposal that will be submitted to the REB in the near future.

17. References

1. Hayashi N, I.T., Yamada T, et al Study on the postprandial blood glucose suppression effect of D-psicose in borderline diabetes and the safety of long-term ingestion by normal human subjects. *Biosci. Biotechnol. Biochem*. **2010**, *74*, 510–519.
2. Heart and Stroke Foundation of Canada, Sugar, heart disease and stroke. Available online: https://www.heartandstroke.ca/-/media/pdf-files/canada/2017-position-statements/sugar-ps-eng.ashx (accessed on 25 August 2017).
3. Diabetes Canada, Diabetes Canada's Position on sugars. Available online: http://www.diabetes.ca/about-cda/public-policy-position-statements/sugars (accessed on 25 August 2017).
4. Malik, V.S., et al., Sugar-sweetened beverages and weight gain in children and adults: a systematic review and meta-analysis. *Am. J. Clin. Nutr.* **2013**, *98*, 1084–102.
5. Imamura, F., et al., Consumption of sugar sweetened beverages, artificially sweetened beverages, and fruit juice and incidence of type 2 diabetes: systematic review, meta-analysis, and estimation of population attributable fraction. *BMJ* **2015**, *351*, h3576.
6. Jayalath, V.H., et al., Sugar-sweetened beverage consumption and incident hypertension: a systematic review and meta-analysis of prospective cohorts. *Am. J. Clin. Nutr.* **2015**, *102*, 914–921.
7. Xi, B., et al., Sugar-sweetened beverages and risk of hypertension and CVD: a dose-response meta-analysis. *Br. J. Nutr.* **2015**, *113*, 709–717.
8. Healthy Kids Panel, No time to wait: The healthy kids strategy. Available online: http://www.health.gov.on.ca/en/common/ministry/publications/reports/healthy_kids/healthy_kids.pdf (accessed on 25 August 2017).
9. Health Canada, Summary of Guiding Principles and Recommendations. Available online: https://www.foodguideconsultation.ca/guiding-principles-summary (accessed on 25 August 2017).
10. Health Canada, Toward Restricting Unhealthy Food and Beverage Marketing to Children Discussion paper for public consultation. Available online: https://s3.ca-central-1.amazonaws.com/ehq-production-canada/documents/attachments/9bced5c3821050c708407be04b299ac6ad286e47/000/006/633/original/Restricting_Marketing_to_Children.pdf (accessed on 25 August 2017).
11. Hsu, W.C., et al., BMI cut points to identify at-risk Asian Americans for type 2 diabetes screening. *Diabetes Care* **2015**, *38*, 150–158.
12. Azad, M.B., et al., Nonnutritive sweeteners and cardiometabolic health: a systematic review and meta-analysis of randomized controlled trials and prospective cohort studies. *CMAJ.* **2017**, *189*, E929–E939.
13. Sievenpiper JL, K.T., Ha V, Viguiliouk E, Auyeung R. The importance of study design in the assessment of non-nutritive sweeteners and cardiometabolic health CMAJ. Published August 4, 2017. Available online: http://www.cmaj.ca/content/189/28/E929.long/reply#cmaj_el_733381 (accessed on August 25, 2017).
14. Suez, J., et al., Artificial sweeteners induce glucose intolerance by altering the gut microbiota. *Nature* **2014**, *514*, 181–186.
15. Frankenfeld, C.L., et al., High-intensity sweetener consumption and gut microbiome content and predicted gene function in a cross-sectional study of adults in the United States. *Ann. Epidemiol.* **2015**, *25*, 736–742.

16. Chang K, Artificial Sweeteners May Disrupt Body's Blood Sugar Controls. The New York Times, September 17, 2014. Available online: https://well.blogs.nytimes.com/2014/09/17/artificial-sweeteners-may-disrupt-bodys-blood-sugar-controls/ (accessed on 25 August 2017).
17. Kirkey S, Fake sugars linked to obesity, heart disease. National Post, July 17, 2017. Available online: http://nationalpost.com/news/0717-na-sugar (accessed on 25 August 2017).
18. Faherty S, Artificial Sweeteners May Have Despicable Impacts on Gut Microbes. Scientific American, Nov 26, 2014. Available online: https://blogs.scientificamerican.com/guest-blog/artificial-sweeteners-may-have-despicable-impacts-on-gut-microbes/ (accessed on 25 August 2017).
19. Sifferlin A, Artificial Sweeteners Are Linked to Weight Gain—Not Weight Loss. Time, July 16 2017. Available online: http://time.com/4859012/artificial-sweeteners-weight-loss/ (accessed on 25 August 2017).
20. Vergano D, Study: Artificial Sweeteners May Trigger Blood Sugar Risks. National Geographic, September 17, 2014. Available online: http://news.nationalgeographic.com/news/2014/09/140917-sweeteners-artificial-blood-sugar-diabetes-health-ngfood/ (accessed on 25 August 2017).
21. Leung W, Sugar substitutes associated with weight gain and health problems, study says. Globe and Mail, July 17, 2017. Available online: https://www.theglobeandmail.com/news/national/sugar-substitutes-linked-to-weight-gain-and-health-problems-study-says/article35704562/ (accessed on 25 August 2017).
22. Brait E, More research needed into the effects sugar substitutes have on health. The Toronto Star, July 17, 2017. Available online: https://www.thestar.com/life/2017/07/17/more-research-needed-into-the-effects-sugar-substitutes-have-on-health.html (accessed on 25 August 2017).
23. Thompson D, Do Artificial Sweeteners Raise Odds for Obesity? WebMD, July 17, 2017. Available online: http://www.webmd.com/food-recipes/news/20170717/do-artificial-sweeteners-raise-odds-for-obesity#1 (accessed on 25 August 2017).
24. Wolever, T.M.S., et al., Effect of adding oat bran to instant oatmeal on glycaemic response in humans - a study to establish the minimum effective dose of oat beta-glucan. *Food. Funct.* **2018**, *9*, 1692–1700.
25. Zeevi, D., et al., Personalized nutrition by prediction of glycemic responses. *Cell* **2015**, *163*, 1079–1094.
26. Alberti, K.G., et al., Harmonizing the metabolic syndrome: a joint interim statement of the International Diabetes Federation Task Force on Epidemiology and Prevention; National Heart, Lung, and Blood Institute; American Heart Association; World Heart Federation; International Atherosclerosis Society; and International Association for the Study of Obesity. *Circulation* **2009**, *120*, 1640–1645.
27. Leung, A., et al., Hypertension Canada's 2017 guidelines for diagnosis, risk assessment, prevention, and treatment of hypertension in adults. *Canadian Journal of Cardiology* **2017**.
28. Kelly, B.J., et al., Power and sample-size estimation for microbiome studies using pairwise distances and PERMANOVA. *Bioinformatics* **2015**, btv183.
29. Suez, J., et al., Artificial sweeteners induce glucose intolerance by altering the gut microbiota. *Nature* **2014**, *514*, 181–186.
30. David, L.A., et al., Diet rapidly and reproducibly alters the human gut microbiome. *Nature* **2014**, *505*, 559–563.
31. Canada, H., Draft Guidance Document on Food Health Claims Related to the Reduction in Post-Prandial Glycaemic Response F.D. Bureau of Nutritional Sciences, Health Products and Food Branch Editor. 2013. p. 12.
32. Food and Drug Administration, Multiple Endpoints in Clinical Trials: Guidance for Industry [Draft Guidance]. 2017.
33. Benjamini, Y. and D. Yekutieli, The control of the false discovery rate in multiple testing under dependency. *Annals of statistics* **2001**, 1165–1188.
34. Benjamini, Y. and Y. Hochberg, Controlling the false discovery rate: a practical and powerful approach to multiple testing. *Journal of the royal statistical society. Series B (Methodological)* **1995**, 289–300.
35. Matsuda, M. and R.A. DeFronzo, Insulin sensitivity indices obtained from oral glucose tolerance testing: comparison with the euglycemic insulin clamp. *Diabetes care* **1999**, *22*, 1462–1470.
36. Dalton, M., et al., Preliminary validation and principal components analysis of the Control of Eating Questionnaire (CoEQ) for the experience of food craving. *Eur. J. Clin. Nutr.* **2015**, *69*, 1313–1317.
37. Heart and Stroke Foundation of Canada, Healthy weight and waist. Available online: https://www.heartandstroke.ca/get-healthy/healthy-weight/healthy-weight-and-waist (accessed on 29 September 2017).
38. Blom, D.J., et al., A 52-week placebo-controlled trial of evolocumab in hyperlipidemia. *N. Engl. J. Med.* **2014**, *370*, 1809–1819.
39. Caporaso, J.G., et al., Ultra-high-throughput microbial community analysis on the Illumina HiSeq and MiSeq platforms. *Isme J.* **2012**, *6*, 1621–1624.
40. Mouzaki, M., et al., Intestinal microbiota in patients with nonalcoholic fatty liver disease. *Hepatology* **2013**, *58*, 120–127.
41. Langille, M.G., et al., Predictive functional profiling of microbial communities using 16S rRNA marker gene sequences. *Nat. Biotechnol.* **2013**, *31*, 814–821.
42. Kadish, A.H. and D.A. Hall, A new method for the continuous monitoring of blood glucose by measurement of dissolved oxygen. *Clinical chemistry* **1965**, *11*, 869–875.
43. Mercodia, Mercodia insulin ELISA. 2008-2017: Uppsala, Sweden.
44. Wolever, T.M., et al., The glycemic index: methodology and clinical implications. *The American journal of clinical nutrition* **1991**, *54*, 846–854.

45. Phillips, D., et al., Understanding oral glucose tolerance: comparison of glucose or insulin measurements during the oral glucose tolerance test with specific measurements of insulin resistance and insulin secretion. *Diabetic Medicine* **1994**, *11*, 286–292.
46. Logue, C., et al., Application of Liquid Chromatography-Tandem Mass Spectrometry To Determine Urinary Concentrations of Five Commonly Used Low-Calorie Sweeteners: A Novel Biomarker Approach for Assessing Recent Intakes? *J. Agric. Food Chem.* **2017**, *65*, 4516–4525.
47. Theytaz, F., et al., Metabolic fate of fructose ingested with and without glucose in a mixed meal. *Nutrients* **2014**, *6*, 2632–2649.
48. Wolever, T.M.S., et al., Effect of serving size and addition of sugar on the glycemic response elicited by oatmeal: A randomized, cross-over study. *Clin. Nutr. ESPEN.* **2016**, *16*, 48–54.
49. Brisbois, T.D., et al., Estimated intakes and sources of total and added sugars in the Canadian diet. *Nutrients* **2014**, *6*, 1899–1912.
50. Singh, G.M., et al., Estimated Global, Regional, and National Disease Burdens Related to Sugar-Sweetened Beverage Consumption in 2010. *Circulation* **2015**, *132*, 639–666.
51. Bleich, S.N., et al., Increasing consumption of sugar-sweetened beverages among US adults: 1988–1994 to 1999—2004. *The American journal of clinical nutrition* **2008**, 26883.
52. Dias, A.G., et al., Genetic variation in putative salt taste receptors and salt taste perception in humans. *Chem. Senses.* 2013, *38*, 137–145.
53. Eny, K.M., et al., Genetic variant in the glucose transporter type 2 is associated with higher intakes of sugars in two distinct populations. *Physiol. Genomics*, **2008**, *33*, 355–360.
54. Wolf, S.M., et al., Managing incidental findings in human subjects research: analysis and recommendations. *J. Law Med. Ethics* **2008**, *36*, 219–48, 211.

Disclaimer/Publisher's Note: The statements, opinions and data contained in all publications are solely those of the individual author(s) and contributor(s) and not of MDPI and/or the editor(s). MDPI and/or the editor(s) disclaim responsibility for any injury to people or property resulting from any ideas, methods, instructions or products referred to in the content.
